# The pump, the exchanger, and the Holy Spirit: tracing the 40-year evolution of the Ouabain-Na^+^ pump endocrine system concept

**DOI:** 10.1097/MS9.0000000000003438

**Published:** 2025-05-30

**Authors:** Chukwuka Elendu, Dependable C. Amaechi, Tochi C. Elendu, Emmanuel C. Amaechi, Ijeoma D. Elendu, Abolore Aminat Ajakaye, Esther S. Ubi, Victor I. Ikejig, Kosisochukwu T. Okwunweze, Ayi T. Debua, John A. Eze, Emmanuel J. Ugwu

**Affiliations:** a Federal University Teaching Hospital, Owerri, Nigeria; b Igbinedion University, Okada, Nigeria; c Imo State University, Owerri, Nigeria; d Madonna University, Elele, Nigeria; e Bogomolets National Medical University, Kyiv, Ukraine; f University of Calabar, Calabar, Nigeria; g Ternopil National Medical University, Ternopil, Ukraine; h Life Support and Medical Centre, Lagos, Nigeria; i University of Calabar Teaching Hospital, Calabar, Nigeria; j Chukwuemeka Odumegwu Ojukwu University, Awka, Nigeria

**Keywords:** endocrine system, hypertension, intracellular signaling, Na^+/^K^+^ pump, Ouabain

## Abstract

The discovery and subsequent evolution of the Ouabain-Na^+^/K^+^ pump endocrine system have profoundly impacted our understanding of cellular physiology and disease mechanisms. Initially identified as a cardiotonic steroid with potent effects on the Na^+^/K^+^ ATPase, Ouabain has been implicated in various physiological and pathological processes. The Na^+^/K^+^ pump, a crucial component of cellular physiology, maintains electrochemical gradients essential for nerve impulse transmission, muscle contraction, and cellular volume regulation. Since Jens Christian Skou’s Nobel Prize-winning discovery in 1957, research has unveiled its broader role in cellular homeostasis and disease. A significant breakthrough was the identification of Ouabain as an endogenous ligand of the Na^+^/K^+^ pump, transforming the pump’s role from a mere ion transporter to a receptor within a hormonal signaling pathway. This discovery has linked the Na^+^/K^+^ pump to complex intracellular signaling pathways, with implications for hypertension, heart failure, and cancer. However, emerging evidence suggests that its role extends beyond cardiovascular dysfunction to neurological disorders such as epilepsy, Alzheimer’s disease, and Parkinson’s disease. In epilepsy, dysregulation of the Na^+^/K^+^ pump contributes to altered ion homeostasis and hyperexcitability. At the same time, in Alzheimer’s disease, its dysfunction has been associated with disrupted calcium signaling, oxidative stress, and amyloid-beta accumulation. Similarly, alterations in Na^+^/K^+^ pump activity have been linked to dopaminergic neuron vulnerability in Parkinson’s disease. This paradigm shift offers exciting therapeutic possibilities for neurodegenerative and neuropsychiatric disorders, including schizophrenia and depression, redefining the pump’s significance across multiple disciplines of medicine.

## Public summary

Scientists once thought that Ouabain, a substance known to affect heart function, only came from plants. However, with the help of advanced laboratory techniques, they discovered that the human body also makes it naturally. Using powerful tools that can identify tiny amounts of chemicals in blood and tissues, researchers confirmed that Ouabain is produced inside the body and may play an essential role in regulating blood pressure and cell function.

**HIGHLIGHTS**

- Ouabain acts as an endogenous hormone regulating the Na⁺/K⁺ pump.
- Na⁺/K⁺ pump is central to signaling in immunity, metabolism, and cancer.
- The Na⁺/K⁺ pump emerges as a master switch in health and disease control.

## Introduction and background

The Na^+^/K^+^ pump, also known as the sodium-potassium ATPase, is a crucial component of cellular physiology and is responsible for maintaining electrochemical gradients across the plasma membrane. This gradient is essential for nerve impulse transmission, muscle contraction, and cellular volume regulation^[1^-^3]^. The Na^+^/K^+^ pump actively transports sodium (Na^+^) ions out of the cell and potassium (K^+^) ions into the cell, utilizing ATP hydrolysis as an energy source. This function is vital for sustaining resting membrane potential and cellular excitability. Beyond its role in ion transport, emerging evidence suggests that the Na^+^/K^+^ pump participates in intracellular signaling pathways^[4-6]^. Ouabain, a cardiac glycoside traditionally associated with heart failure treatment, is a key factor in this expanded role. Ouabain inhibits the Na^+^/K^+^ pump by binding to its extracellular domain, increasing intracellular sodium concentration and subsequent alterations in cellular function. The recognition that Ouabain is an endogenous hormone has led to the hypothesis that the Na^+^/K^+^ pump functions within an endocrine system, influencing ion homeostasis and blood pressure regulation^[7]^. The interaction between Ouabain and the Na^+^/K^+^ pump triggers intracellular signaling cascades involving mitogen-activated protein kinases (MAPKs), phosphoinositide 3-kinase (PI3K), and reactive oxygen species (ROS). These pathways are implicated in processes such as cell proliferation, apoptosis, and vascular tone regulation^[8-10]^. Liquid chromatography-mass spectrometry (LC-MS) is one of the methods employed in detecting endogenous Ouabain; it allows the precise separation, identification, and quantification of small molecules in biological samples. LC-MS has been particularly valuable due to its high sensitivity and specificity, enabling researchers to detect Ouabain at nanomolar concentrations in plasma, urine, and tissue extracts^[2,3]^. High-performance liquid chromatography (HPLC) has also played a crucial role in isolating and purifying Ouabain from biological matrices before mass spectrometric analysis, ensuring accurate identification. Electrospray ionization mass spectrometry (ESI-MS) has further contributed to the confirmation of Ouabain as an endogenous compound by facilitating the detection of its molecular signature with high precision^[4,5]^. This technique allows for the ionization of molecules in solution without excessive fragmentation, preserving the integrity of the compound and making it easier to distinguish Ouabain from structurally similar molecules. Additionally, immunoassays such as enzyme-linked immunosorbent assays (ELISA) have been used to detect Ouabain-like compounds in biological fluids, providing further evidence of their endogenous presence^[6,7]^. However, immunoassays alone may lack the specificity to differentiate Ouabain from other cardiotonic steroids, making mass spectrometric validation essential. Combining these techniques has provided compelling evidence that Ouabain is not merely an exogenous cardiac glycoside but an endogenous regulator with a significant role in cellular signaling and ion homeostasis (Table 1). By employing these advanced methodologies, researchers have tracked fluctuations in endogenous Ouabain levels in response to physiological and pathological conditions, strengthening the understanding of its role in health and disease. Recent epidemiological studies highlight the increasing global burden of hypertension and heart failure. According to the 2023 Global Burden of Disease report, hypertension affects over 1.4 billion people worldwide, with a prevalence exceeding 30% in adults. Additionally, heart failure remains a leading cause of morbidity and mortality, impacting over 64 million individuals globally^[11-13]^. These findings underscore the urgent need to understand the molecular mechanisms underlying cardiovascular regulation, including the role of the Ouabain-Na^+^/K^+^ pump endocrine system. Dysregulation of the Na^+^/K^+^ pump’s activity and Ouabain signaling has been linked to various diseases, including hypertension, cardiovascular disorders, neurological conditions, and cancer. Furthermore, alterations in Ouabain levels have been associated with renal sodium handling and conditions like chronic kidney disease and salt-sensitive hypertension^[14-16]^. The evolving understanding of the Na^+^/K^+^ pump as more than an ion transporter has significant research and therapeutic development implications. Investigating its role in cellular signaling and systemic regulation provides insight into disease mechanisms and potential treatment strategies for cardiovascular, neurological, and renal disorders^[17]^. Given the expanding role of the Na^+^/K^+^ pump in cellular signaling, disease mechanisms, and therapeutic interventions, our paper aims to trace the origins and 40-year evolution of the Ouabain-Na^+^ pump endocrine system concept. We highlight key milestones (Table 2), shifts in scientific understanding, and current perspectives while emphasizing their physiological and clinical implications. Beyond its well-established physiological roles, emerging research highlights the impact of genetic variations in the Na^+^/K^+^ pump on individual responses to targeted therapies. Genetic polymorphisms in ATP1A1 have been linked to altered Na^+^/K^+^ pump activity, which may modify an individual’s sensitivity to cardiac glycosides like Ouabain and digoxin^[18]^. For instance, certain ATP1A1 variants have been associated with increased binding affinity for Ouabain, leading to enhanced inhibitory effects, while others may confer resistance to cardiotonic steroids^[18]^. These differences could explain interindividual variability in the therapeutic and toxic effects of Na^+^/K^+^ pump inhibitors used in conditions such as heart failure and hypertension. Beyond cardiovascular implications, ATP1A3 mutations have been implicated in neurological disorders such as Alternating Hemiplegia of Childhood (AHC) and rapid onset dystonia-parkinsonism (RDP), conditions characterized by dysfunctional neuronal excitability^[19]^. These mutations impact neuronal Na^+^/K^+^ pump function and alter responses to neuroactive steroids and other pump-modulating therapies. Understanding these genetic variations provides an opportunity for precision medicine approaches, where patients with specific Na^+^/K^+^ pump genotypes could receive tailored treatments to optimize efficacy while minimizing adverse effects^[20]^. Future research should explore how these genetic differences affect endogenous Ouabain signaling and its role in cardiovascular and neurological health. Large-scale genome-wide association studies (GWAS) and functional assays could help elucidate the precise impact of Na^+^/K^+^ pump polymorphisms on disease susceptibility and drug responses. Integrating pharmacogenetic insights into clinical practice could enhance personalized therapeutic strategies for patients receiving Na^+^/K^+^ pump-targeting therapies.

**Table 1:** Analytic detection of EO in mammals

Senior/key author(s) (location)	Year	Source	Material	Analytical method
A. Positive detection of EO in mammals (published spectra/MS and NMR data)				
1. Hamlyn/Blaustein/DuCharme (Baltimore, MD/Kalamazoo, MI)	1991	Human	Plasma	MS
2. Mathews/DuCharme/Hamlyn (Kalamazoo/Baltimore)	1991	Human	Plasma	MS
3. Inagami/Tamura (Nashville, TN)	1994	Rat	Urine	MS, NMR
4. Doris (Lubbock, TX)	1994	Bovine	Adrenal	–a
5. Defaye/Perrin (Grenoble, France)	1997	Bovine	Adrenal	MS
6. Schoner/Schneider (Giessen, Germany)	1998	Bovine	Adrenal	MS, NMR
7. Nakanishi/Kawamura/Haupert (New York, NY/Boston, MA)	1999	Bovine	Hypothalamus	NMR
8. Takahashi/Komiyama (Tokyo, Japan)	2000	Human	Plasma	MS
9. Manunta/Hamlyn/Pitzalis (Milan, Italy/Baltimore)	2005	Human	Plasma	MS
10. Hamlyn/Manunta (Baltimore/Milan)	2006	Human	Plasma	MS
11. Hamlyn/Jacobs (Baltimore/Milan)	2012	Rat	Plasma	MS
12. Hamlyn/Leenen/Blaustein (Baltimore/Ottawa, Canada)	2014	Rat	Plasma	MS
13. Hamlyn/Blaustein (Baltimore)	2016	Rat/Mouse	Plasma	MS
B. Identification of EO isomers (published spectra)				
1. Hamlyn/Jacobs (Baltimore)	2012	Rat	Plasma	MS
2. Hamlyn/Leenen (Baltimore/Ottawa)	2014	Rat	Plasma	MS
C. Did not identify EO in human samples				
1. Doris (Lubbock, TX)	1994	Human	Plasma	RIA^b^
2. Nicholls/Lewis (Christchurch, New Zealand)	1994	Human	Plasma	ELISA^b^
3. Hilton (London, UK)	1996	Human	Placenta	MS
4. Vogeser/Baecher (Munich)	2014	Human	Plasma	MS^c^

^a^Method not specified in the publication (i.e., not clearly stated).

^b^Radioimmunoassay (RIA) or ELISA used, which lack confirmation by structural methods (e.g., MS/NMR).

^c^Study employed MS but did not confirm the presence of EO.Identified by elution pattern; method used was not analytically definitive. The original MS trace had a break near 5.0 min, but later spectra from two patients showed a signal with the same mass/charge ratio as Ouabain, eluting 0.3 min earlier (adapted from Blaustein, 2018).

**Table 2:** Milestones in elucidating the endogenous Ouabain-Na^+^ pump endocrine system

Year	Key Investigator	Discovery
1785	Withering	Digitalis is a therapy for heart failure (HF)
1861	Kirk	Discovery that *S. kombé* seed juice (contains Ouabain) is cardiotonic
1888	Arnaud	Isolation (from ouabaio tree bark) and naming of Ouabain
1953	Schatzmann	Cardiotonic steroids (CTS) inhibit Na^+^ pumps
1953	Szent-Gyorgi	Digitalis may replace a missing endogenous compound
1957	Skou	Na^+^ pump is an Na-K-ATPase (NKA)
1967	Dahl	A humoral factor contributes to salt-dependent hypertension
1968	Reuter	Na/Ca exchanger (NCX) discovery;
1968/9	Baker/Blaustein	NCX discovery: “missing link” explains the cardiotonic effect of CTSs
1973	Reuter/Blaustein	NCX is present in arteries and is postulated to modulate blood pressure (BP)
1975	Bricker	Natriuretic hormone (“third factor”) is a NKA inhibitor
1976	Haddy	Na^+^ pump-endogenous CTS-hypertension hypothesis
1977	Blaustein	Na^+^ pump-NCX-endogenous CTS-hypertension hypothesis
1978	Brody	Sustained hypertension requires the hypothalamic AV3V region
1982	Hamlyn/Blaustein	A circulating NKA inhibitor correlates with BP in humans
1983	Atkinson	Low (≤10–7 M) Ouabain concentrations trigger cell proliferation
1984	Erdmann	Rat cardiac myocytes express ~90% low (α1) and ~10% high (α2) Ouabain affinity Na^+^ pumps
1985–7	Lingrel/Shull	NKA is cloned; 3 catalytic (α) subunit isoforms (α1–α3) identified
1989	Philipson	Ouabain upregulates NCX1 expression in cardiomyocytes
1989	Delva	Plasma Ouabain-like compound (OLC) is elevated in pregnancy and more so in preeclampsia
1991	Hamlyn/Upjohn group/Blaustein	An endogenous CTS in humans identified as “Ouabain” (EO)
1992	Gottlieb/Hamlyn	Plasma EO correlates inversely with cardiac index in HF patients
1993	Haddy/Pamnani/Hamlyn	Ouabain (chronic treatment) causes hypertension in rats
1993	Hamlyn/Manunta	Digoxin doesn’t induce hypertension, but antagonizes Ouabain’s effect
1993/4	Hamlyn	Adrenal glands synthesize and secrete EO
1994	EP and CE Gomez-Sanchez	Active anti-Ouabain immunization attenuates DOCA-salt hypertension
1994	Leenen	“Brain Ouabain” mediates salt-sensitive hypertension
1995	Hollenberg	Fab fragments (Digibind) attenuate DOCA + salt hypertension in rats
1995	Leenen	“Brain Ouabain” mediates sympathetic hyperactivity in HF
1995	Rossi/Manunta/Hamlyn Hamilton	~50% of patients with essential hypertension or aldosterone-producing adenomas have elevated plasma EO that correlates with mean BP
1996–8	Askari/Xie	Ouabain binding to NKA activates protein kinase (PK) signaling cascades
1997–8	Defaye/Perrin	[^14^C]rhamnose is incorporated into EO in bovine adrenocortical cells
1997–8	Bianchi/Ferrari	Synthesis of rostafuroxin (PST2238), an Ouabain antagonist but not NKA inhibitor, attenuates Ouabain-induced hypertension
1997–8	Blaustein/Juhaszova	α2 Na^+^ pumps and NCX1 colocalize at PLasmERosomes, and help regulate local [Ca^2+^]_CYT_ and Ca^2+^signaling
2000	Xie/Askari	Ouabain activates PK cascades independent of changes in [Na^+^] or [Ca^2+^]
2005	Lingrel	ACTH-hypertension requires α2 NKA Ouabain binding sites and EO
2006	Hamlyn/Manunta/Hamilton	Plasma EO in humans is elevated by both high salt and salt depletion
2008	Lingrel/Lorenz	Digibind prevents natriuresis promoted by high Ouabain affinity renal α1 NKA*
2009	Lichtstein	Anti-Ouabain antibodies promote adrenal cortex, not medulla, hypertrophy
2009	Heiny/Lingrel	α2 NKA Ouabain binding sites modulate skeletal muscle fatigue*
2010	Aperia	Ouabain rescues fetal kidney development in malnourished pregnant rats*
2010	Golovina	Arterial Ca^2+^ transporters are upregulated in Ouabain- and salt-sensitive hypertension
2010	Leenen	A novel hypothalamic slow neuromodulatory pathway [Ang II-AT_1_R-aldosterone-MR-ENaC-EO-(α2) Na^+^ pumps] sustains BP in hypertension*
2011	Lorenz/Lingrel	Pressure overload-induced cardiac hypertrophy and dysfunction requires Ouabain-sensitive Na^+^ pumps and EO
2011	Van Huysse/Lingrel	NaCl-induced hypertension requires α2 NKA Ouabain binding sites and EO
2011	Vorhees/Williams/Lingrel	Mice with Ouabain-resistant α2 NKA exhibit specific behavioral abnormalities and learning impairment*
2012	Hamlyn	MS shows EO is elevated in pregnant rats; detection of two EO isomers in rat plasma
2013	Golovina/Hamlyn	Ouabain, not digoxin, upregulates arterial Ca^2+^ transporters and elevates BP via a Src-dependent mechanism; first evidence that an ion transporter can act as a biased hormone receptor
2013	Manunta/Gottlieb/Bianchi/Hamlyn	Preoperative plasma EO predicts acute kidney injury (AKI) in cardiac surgery patients; renal function is impaired in Ouabain-hypertensive rats
2014	Ferrandi/Ferrari	Rostafuroxin attenuates Ouabain-induced high BP and kidney injury
2014	Leenen/Hamlyn/Blaustein	A hypothalamic slow neuromodulatory pathway regulates plasma EO
2015	Lichtstein	In pregnant rats, anti-Ouabain antibodies reduce fetal birth weight, and impair fetal kidney and liver development but increase cardiac growth*
2017	Scavone	α2 NKA silencing abolishes the anti-inflammatory and anti-apoptotic effects of nanomolar Ouabain in cultured glial cells
2018	Blaustein	α2 NKA Ouabain binding sites modulate basal BP*

These data highlight the role of the EO-α2 Na⁺ pump endocrine system in regulating key physiological processes, including blood pressure, exercise endurance, fetal organ development, natriuresis, sympathetic activity, and cognitive function [adapted from Blaustein, 2018].

## Information analyzing procedures

We used a systematic search strategy across multiple scientific databases, including PubMed, Google Scholar, Scopus, and Web of Science. Keywords such as “Na^+^/K^+^ pump,” “ouabain,” “endocrine system,” “hypertension,” “intracellular signaling,” and “cardiovascular diseases” were used to identify relevant articles. The search was restricted to articles published in English and included primary research studies, review articles and meta-analyses. The literature search was further refined by focusing on research published from 1980 to the present, reflecting the timeline of significant developments in the field. Inclusion criteria for selecting articles included studies that specifically addressed the role of the Na^+^/K^+^ pump in cellular physiology, the discovery and characterization of Ouabain as an endogenous ligand, and the evolving understanding of the Na^+^/K^+^ pump as part of an endocrine system. Articles also discussed the molecular mechanisms, signaling pathways, and clinical implications of Na^+^/K^+^ pump modulation. Exclusion criteria involved studies that did not directly relate to the Na^+^/K^+^ pump or Ouabain, studies with inconclusive or poorly defined outcomes, and those published in non-peer-reviewed sources. The analysis involved qualitatively synthesizing the gathered literature and identifying key themes and trends in understanding the Na^+^/K^+^ pump and its broader implications. Specific attention was given to the controversies surrounding the endogenous nature of Ouabain, the physiological and pathophysiological roles of the Na^+^/K^+^ pump, and its potential as a therapeutic target. Data extraction was performed by summarizing the findings of individual studies, with a focus on the mechanisms of action, clinical implications, and potential therapeutic applications of Na^+^/K^+^ pump modulation.

## Historical context

**Figure 1.**
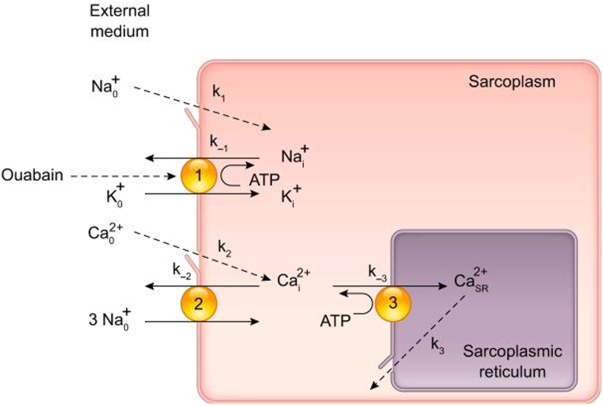
Early conceptual model of Na⁺-driven Ca^2^⁺ regulation and vascular tone in arterial smooth muscle (1977). Block diagram (1977 version) illustrating the proposed model of Ca^2^⁺ regulation in arterial smooth muscle cells. It shows how Na⁺ pumps, Na⁺/Ca^2^⁺ exchangers (NCX), and an endogenous Ouabain-like compound (OLC) may regulate intracellular Ca^2^⁺ and vascular tone. Key elements include the Ouabain-sensitive Na⁺ pump, NCX (then thought to mediate Ca^2^⁺ extrusion), and the SR Ca^2^⁺ pump (SERCA). Na⁺ and Ca^2^⁺ primarily enter via voltage-gated channels (k1, k2), and Ca^2^⁺ is released from the SR via ryanodine or IP_3_ receptors (k3). This model predates the identification of Na⁺ pump isoforms and PLasmERosomes. Source: Blaustein^[1]^.

The Na^+^/K^+^ pump was first identified in the late 1950s by Danish scientist Jens Christian Skou, who was awarded the Nobel Prize in Chemistry in 1997 for this groundbreaking work. Skou’s discovery was initially met with skepticism, but it eventually revolutionized the field of biochemistry and physiology. He demonstrated that the enzyme responsible for this ion exchange was an ATPase, meaning that it used the energy derived from ATP hydrolysis to drive the active transport of ions against their concentration gradients^[10-12]^. This was a significant finding because it provided the first clear evidence of an enzyme that could couple the energy from ATP hydrolysis directly to the transport of ions, a process now recognized as fundamental to cell function. Before Skou’s discovery, the movement of ions across cell membranes was a poorly understood phenomenon. Cells were known to maintain high concentrations of K^+^ and low concentrations of Na^+^ relative to the extracellular environment, but the mechanisms responsible for this distribution were unclear. Skou’s work demonstrated that the Na^+^/K^+^ pump actively transported Na^+^ out of the cell and K^+^ into the cell, thereby creating and maintaining the ion gradients that are crucial for numerous cellular processes, including the generation of action potentials in nerve and muscle cells^[2,4,10]^. The Na^+^/K^+^ pump functions by cycling through a series of conformational changes that allow it to bind and transport ions. In its E1 conformation, the pump has a high affinity for Na^+^ ions and binds three from the intracellular side. Upon binding ATP, the pump undergoes phosphorylation, which leads to a conformational change to the E2 state, releasing Na^+^ ions into the extracellular space. This change in conformation also increases the pump’s affinity for K^+^ ions, allowing it to bind two K^+^ ions from the extracellular side. The subsequent dephosphorylation of the pump returns it to the E1 state, releasing the K^+^ ions into the cell and completing the cycle^[3,16,21]^. This cyclical process is essential for maintaining the ion gradients and driving other transport processes in the cell. The importance of the Na^+^/K^+^ pump extends beyond its role in maintaining ion gradients. It is also crucial for regulating cell volume and osmotic balance. Cells must continuously counteract the osmotic pressure that would otherwise lead to swelling and lysis, and the Na^+^/K^+^ pump achieves this by extruding Na^+^ ions, which are osmotically active. By maintaining a low intracellular concentration of Na^+^, the pump reduces the osmotic influx of water, thus stabilizing cell volume^[4-6]^. In the years following Skou’s discovery, the Na^+^/K^+^ pump was extensively studied, and its role in various physiological processes became increasingly clear. It was found to be particularly important in excitable tissues such as nerves and muscles, where the rapid and precise regulation of ion gradients is essential for function. In neurons, for example, the Na^+^/K^+^ pump helps to reset the membrane potential after an action potential, thereby preparing the neuron for the next signal^[5]^. In the heart, the pump is critical for maintaining the ion gradients that underlie the rhythmic contractions of cardiac muscle^[6,10,17]^. As research into the Na^+^/K^+^ pump progressed, it became evident that this enzyme was not just a simple ion transporter but a complex protein with multiple regulatory mechanisms. Various factors, including intracellular levels of Na^+^ and K^+^, phosphorylation, and interaction with other proteins and signaling molecules, regulate the activity of the Na^+^/K^+^ pump. This regulation allows the pump to respond to changes in the cellular environment and to modulate its activity according to the needs of the cell^[7]^. One of the most intriguing aspects of the Na^+^/K^+^ pump’s regulation is its interaction with endogenous and exogenous compounds that can modulate its activity. Among these, Ouabain stands out as a vital molecule. Ouabain is a cardiac glycoside that was historically used in traditional medicine to treat heart failure. It was first isolated from the plant *Strophanthus gratus* and has been used in various forms in African and Western medicine for centuries. The introduction of Ouabain into scientific research provided new insights into the functioning of the Na^+^/K^+^ pump and opened up new avenues for understanding its role in physiology and disease^[8,19,22]^. The connection between Ouabain and the Na^+^/K^+^ pump was first established in the 1950s when researchers observed that Ouabain could inhibit the activity of the Na^+^/K^+^ pump. This inhibition occurs because Ouabain binds specifically to the extracellular surface of the Na^+^/K^+^ pump, stabilizing the enzyme in its E2 conformation and preventing the dephosphorylation step necessary to release K^+^ ions into the cell^[9]^. As a result, the pump cannot complete its cycle, leading to a buildup of intracellular Na^+^ and a decrease in intracellular K^+^. The discovery that Ouabain could inhibit the Na^+^/K^+^ pump had significant implications for understanding the pump’s function and the mechanism of action of cardiac glycosides. It was found that the inhibition of the Na^+^/K^+^ pump by Ouabain and other cardiac glycosides could increase the force of cardiac muscle contraction, making these compounds useful in treating heart failure. The increased intracellular Na^+^ concentration that results from pump inhibition leads to an increased intracellular Ca^2+^ concentration via the Na^+^/ Ca^2+^ exchanger, which enhances cardiac contractility^[5,10,23]^. In 1977, these emerging insights into ion regulation culminated in one of the earliest integrative models of calcium homeostasis and vascular tone in arterial smooth muscle cells. This model – shown in Fig. 1 – illustrates how the Ouabain-sensitive Na⁺ pump, the Na⁺/Ca^2^⁺ exchanger (NCX), and an endogenous Ouabain-like compound (OLC) might cooperate to regulate intracellular Ca^2^⁺ levels and vascular tone. Although the model predates the discovery of Na⁺ pump isoforms and PLasmERosomes, it marked a significant step forward in conceptualizing the pump-exchanger-SR interplay as a dynamic regulatory axis. Identifying Ouabain as an inhibitor of the Na^+^/K^+^ pump also raised important questions about the physiological role of this interaction. Researchers began to investigate whether Ouabain or similar compounds could be produced endogenously in the human body and whether they might regulate the activity of the Na^+^/K^+^ pump under normal physiological conditions. This line of inquiry led to the discovery of endogenous cardiac glycosides, including Ouabain itself, which were found to be produced by the adrenal glands and hypothalamus^[11-13]^. The discovery of endogenous Ouabain and its role in regulating the Na^+^/K^+^ pump has had far-reaching implications for understanding various physiological processes and diseases. Endogenous Ouabain is now recognized as a hormone regulating blood pressure, electrolyte balance, and cardiovascular function. It has been shown to modulate the activity of the Na^+^/K^+^ pump in response to changes in blood volume, sodium intake, and other factors, thereby helping to maintain homeostasis^[12,14,21]^. In addition to its role in normal physiology, endogenous Ouabain has been implicated in the pathogenesis of several diseases, including hypertension, heart failure, and kidney disease. Elevated levels of endogenous Ouabain have been found in patients with these conditions, and it is believed that the dysregulation of Ouabain production or the Na^+^/K^+^ pump’s response to Ouabain may contribute to the development and progression of these diseases^[13]^. Identifying endogenous Ouabain as a potential biomarker for cardiovascular diseases has opened up new possibilities for diagnosis and treatment, and ongoing research continues to explore its therapeutic potential. Over the past several decades, studying the Na^+^/K^+^ pump and its interaction with Ouabain has expanded to include various disciplines, from molecular biology and pharmacology to clinical medicine. The elucidation of the molecular structure of the Na^+^/K^+^ pump, along with advances in techniques such as X-ray crystallography and cryo-electron microscopy, has provided detailed insights into how Ouabain binds to and inhibits the pump at the atomic level^[21]^. These structural studies have deepened the understanding of the pump’s function and informed the design of new drugs that target the Na^+^/K^+^ pump to treat various diseases.

## Development of the endocrine system concept

The early days of the Na^+^/K^+^ pump research focused on its role in maintaining the electrochemical gradients essential for nerve impulses and muscle contractions. However, as scientists delved deeper into the regulatory mechanisms of this pump, it became clear that its activity was not solely governed by the immediate ionic needs of the cell but was also subject to hormonal control. This marked the beginning of a shift in understanding the pump’s role from merely a cellular housekeeping tool to a critical player in the broader endocrine system^[10-12]^. One of the key hormones implicated in regulating the Na^+^/K^+^ pump is aldosterone, a steroid hormone produced by the adrenal cortex. Aldosterone plays a crucial role in regulating sodium and potassium balance in the body by increasing the activity of the Na^+^/K^+^ pump, particularly in the kidneys. Through this mechanism, aldosterone promotes sodium reabsorption and potassium excretion, thereby contributing to the regulation of blood pressure and fluid balance^[13,14,21]^. The discovery of aldosterone’s effects on the Na^+^/K^+^ pump was one of the first indications that this pump could be integrated into the endocrine system, linking it to the broader physiological processes that regulate homeostasis. In addition to aldosterone, other hormones influence the Na^+^/K^+^ pump. Insulin, for example, was shown to increase the activity of the Na^+^/K^+^ pump in various tissues, including skeletal muscle and adipocytes. Insulin’s effect on the Na^+^/K^+^ pump is significant in glucose uptake, as the pump’s activity helps maintain the ionic gradients necessary for glucose transport into cells. This connection between insulin and the Na^+^/K^+^ pump further solidified the idea that the pump was more than just an ion transporter; it was a crucial component of the endocrine regulation of metabolism^[2,15,16]^. Identifying endogenous Ouabain as a hormone that could modulate the Na^+^/K^+^ pump marked a significant turning point in developing the endocrine system concept. Endogenous Ouabain was initially identified in the 1980s, and its presence in the human body challenged the traditional view of cardiac glycosides as merely exogenous compounds derived from plants. The realization that the body produces its own Ouabain-like compounds opened up new avenues of research into how the Na^+^/K^+^ pump is regulated under physiological and pathological conditions^[3,17,18]^. Endogenous Ouabain is now recognized as a key player in regulating cardiovascular function. The adrenal glands primarily produce it, and it has been shown to influence blood pressure by modulating the activity of the Na^+^/K^+^ pump in vascular smooth muscle cells and other tissues. By inhibiting the Na^+^/K^+^ pump, endogenous Ouabain can increase intracellular sodium levels, increasing intracellular calcium via the Na^+^/ Ca^2+^ exchanger. This rise in calcium enhances vascular tone and contractility, contributing to the regulation of blood pressure^[4,19,20]^. The concept of the Na^+^/K^+^ pump as part of an endocrine system gained further traction with the discovery of the Ouabain-binding site on the pump itself. This site was highly specific, suggesting that the interaction between Ouabain and the Na^+^/K^+^ pump was not just a byproduct of evolutionary coincidence but a highly regulated process. The binding of Ouabain to its site on the Na^+^/K^+^ pump inhibits the pump’s activity. Still, it also triggers a cascade of intracellular signaling events that can affect various cellular functions, including gene expression, apoptosis, and cell proliferation^[5,22,24]^. The idea that the Na^+^/K^+^ pump could serve as both an ion transporter and a receptor for signaling molecules like Ouabain was a radical departure from the traditional view of membrane proteins as either transporters or receptors, but not both. This dual role of the Na^+^/K^+^ pump fits well within the framework of the endocrine system, where hormones often have multiple, interconnected effects on different tissues and organs. The concept of the Na^+^/K^+^ pump as part of an endocrine system has since been expanded to include its interactions with other hormones and signaling molecules, further highlighting its importance in physiological regulation^[5,6,25]^. In recent years, the concept of the endocrine system associated with the Na^+^/K^+^ pump has been refined and expanded by studying its interactions with other cellular signaling pathways. For example, the interaction between the Na^+^/K^+^ pump and the Src kinase signaling pathway is critical for regulating various cellular functions. Src kinase is a non-receptor tyrosine kinase that is activated by the binding of Ouabain to the Na^+^/K^+^ pump. Once activated, Src kinase can phosphorylate a variety of downstream targets, leading to changes in cell adhesion, migration, and survival^[7,23]^. The cross-talk between the Na^+^/K^+^ pump and the Src kinase signaling pathway illustrates how the pump can integrate extracellular signals (such as those from Ouabain) with intracellular signaling networks, thereby coordinating a wide range of cellular responses. This ability of the Na^+^/K^+^ pump to function as both a transporter and a signal transducer underscores its role as a key component of the endocrine system, with implications for a variety of physiological and pathological processes^[8,26,27]^. The endocrine system concept of the Na^+^/K^+^ pump has also been applied to studying various diseases beyond cardiovascular dysfunction, including neurological disorders such as epilepsy, Alzheimer’s disease, and Parkinson’s disease. Emerging evidence suggests that the Na^+^/K^+^ pump regulates neuronal excitability and synaptic plasticity. Dysregulation of this pump has been implicated in epilepsy, where altered ion homeostasis contributes to hyperexcitability. Similarly, in Alzheimer’s disease, the Na^+^/K^+^ pump’s dysfunction has been linked to disrupted calcium signaling, oxidative stress, and amyloid-beta accumulation, all of which contribute to neurodegeneration^[6,8,9]^. In Parkinson’s disease, alterations in the Na^+^/K^+^ pump activity have been associated with dopaminergic neuron vulnerability, suggesting a role in disease progression^[10,11]^. The broader implications of the Na^+^/K^+^ pump’s involvement in neurodegenerative diseases underscore the need for further research into its therapeutic potential. The potential therapeutic applications of the endocrine system concept of the Na^+^/K^+^ pump extend beyond hypertension and heart failure to neurodegenerative and neuropsychiatric disorders. Given its involvement in neuronal signaling, targeting the Na^+^/K^+^ pump could offer new treatment avenues for epilepsy, depression, schizophrenia, and neurodegenerative conditions. The cross-talk between the Na^+^/K^+^ pump, endogenous Ouabain, and other signaling molecules highlights its role in maintaining homeostasis at both the cellular and systemic levels. Future studies focusing on the genetic and epigenetic regulation of the Na^+^/K^+^ pump may provide personalized therapeutic approaches for conditions where pump dysregulation is implicated^[12,13,21]^.

## Mechanistic pathways

**Figure 2.**
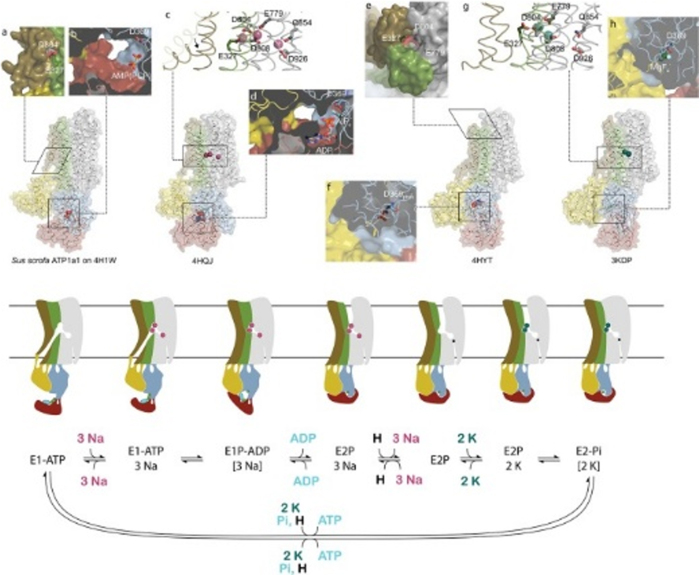
Structural dynamics and conformational transitions during the sodium pump catalytic cycle. Illustration of the conformational transitions during the Na⁺/K⁺-ATPase catalytic cycle. The diagram features three experimentally determined structures and one homology model, each aligned with the corresponding steps of the pump cycle, depicted below through both schematic cartoons and a reaction sequence. Eight labeled insets (a–h) emphasize key structural features at various stages. The homology model of the pig α1 subunit, based on the SERCA E1-ATP state (PDB ID: 4H1W), demonstrates an inward-facing conformation granting access to the ion-binding sites, highlighted by residues D804 and E327 (a). At this stage, the ATP analog AMPPCP binds with only the β- and γ-phosphates resolved, indicating a non-reactive orientation relative to D369 (b). Upon sodium binding, TM1 undergoes conformational rearrangement that obstructs the cytoplasmic entry path (c), while the cytoplasmic domains close in on the nucleotide, enabling interaction with D369 (d). Subsequent occlusion of sodium and ADP release facilitates the opening of an extracellular pathway for sodium ion exit. The outward-facing conformation, modeled after the Ouabain-bound structure (PDB ID: 4HYT, shown here without Ouabain), reveals three ion-coordinating residues accessible from the extracellular space €, with complete encasement of phosphorylated D369 by the cytoplasmic domains (f). Binding of two potassium ions at the extracellular side (g) promotes gate closure and initiates dephosphorylation of D369 (h). A narrow cytoplasmic pathway illustrated in the cartoon representation reflects a proposed C-terminal proton channel responsible for maintaining charge balance. Structural color scheme corresponds to Fig. 8. Source: Adapted from Clausen MV, Hilbers F, Poulsen H. Front Physiol. 2017;8:371. Doi:10.3389/fphys.2017.00371.

**Figure 3.**
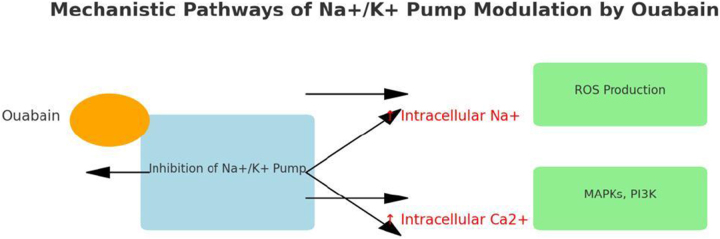
Mechanistic pathways of Ouabain-Na^+^/K^+^ pump interaction and signal transduction. Here’s a schematic diagram illustrating the mechanistic pathways of Ouabain’s modulation of the Na^+^/K^+^ pump. The diagram shows how Ouabain binding to the Na^+^/K^+^ pump leads to its inhibition, resulting in increased intracellular sodium (Na^+^) and calcium (Ca^2+^) levels, which subsequently activate reactive oxygen species (ROS) production and various intracellular signaling pathways like MAPKs and PI3K. Source: Authors’ creations.

**Figure 4.**
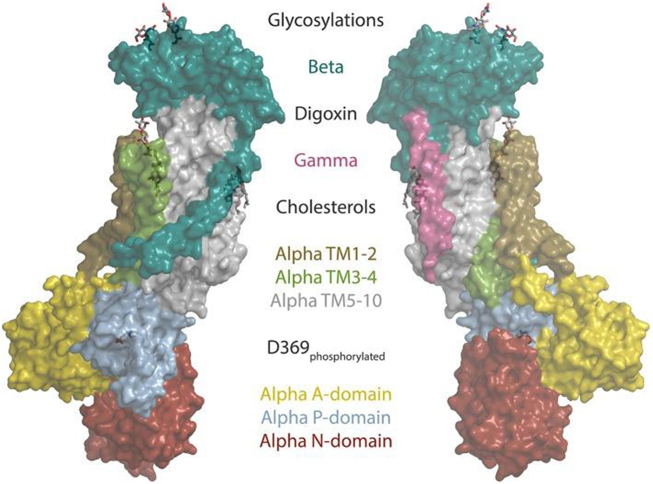
Structural architecture of the sodium pump bound to digoxin. structural model of the Na⁺/K⁺-ATPase (α1 isoform) with digoxin bound, highlighting key subunits, domains, and ligands including glycosylations, cholesterol, and phosphorylated D369. Adapted from Clausen et al. 2017 (PDB: 4RET).

**Figure 5.**
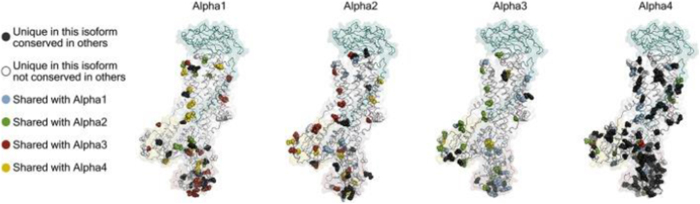
Homology models of human Na⁺/K⁺-ATPase alpha isoforms highlighting structural variations. Homology models representing the four human Na⁺/K⁺-ATPase alpha isoforms, constructed based on the potassium-occluded crystal structure 3 KDP. Isoform-specific amino acid variations are highlighted as spheres. To focus on meaningful structural differences, conservative substitutions were excluded – amino acids were considered functionally equivalent within the following groups: (L, I, V), (E, D), (K, R), (Q, N), (S, T), (Y, F), and (M, C). Residues such as H, G, P, A, and W were treated individually and not grouped. Adapted from Clausen MV, Hilbers F, Poulsen H. Front Physiol. 2017;8:371. Doi:10.3389/fphys.2017.00371.

The pump operates through an ATP-dependent mechanism, wherein ATP hydrolysis drives the conformational changes necessary to translocate ions across the plasma membrane (Fig. 2). The binding of Ouabain to the Na^+^/K^+^ pump represents a pivotal event in the modulation of the pump’s activity (see Fig. 3). Ouabain, a cardiac glycoside traditionally used to treat heart failure, inhibits the Na^+^/K^+^ pump by binding to its extracellular domain. This inhibition increases intracellular sodium levels, affecting various cellular processes^[3,4]^. The precise binding site of Ouabain on the Na^+^/K^+^ pump has been identified through crystallographic studies, which reveal that Ouabain binds to the α-subunit of the pump at a site near the extracellular surface of the membrane^[5]^. The structural basis of this interaction is illustrated in Fig. 4, showing the pig α1 isoform with digoxin (a close analog of Ouabain) bound at the extracellular site. This binding stabilizes the pump in its E2-P conformation, preventing the dephosphorylation required for the pump to return to its E1 state and continue the ion transport cycle. Consequently, the pump’s ability to extrude Na^+^ and import K^+^ is compromised, leading to altered cellular ion homeostasis^[6,7]^. The increase in intracellular sodium concentration resulting from Na^+^/K^+^ pump inhibition has far-reaching consequences for cellular function. One of the most immediate effects is altering the electrochemical gradient across the plasma membrane, which is critical for maintaining the resting membrane potential and the excitability of nerve and muscle cells^[8]^. The disruption of this gradient can lead to depolarization of the membrane, affecting the propagation of action potentials in excitable tissues such as the heart and nervous system and leading to altered function^[9]^. The impact of Na^+^/K^+^ pump inhibition on the heart is particularly significant, as it can lead to positive inotropy, an increase in the force of cardiac muscle contraction. This effect is beneficial in treating heart failure, where increased contractility can improve cardiac output. However, prolonged inhibition of the Na^+^/K^+^ pump can also lead to adverse effects, such as arrhythmias, due to the disruption of ion homeostasis^[10]^. Beyond its role in ion transport, the Na^+^/K^+^ pump has been implicated in various signaling pathways modulated by Ouabain binding. One of the key signaling mechanisms involves the activation of the mitogen-activated protein kinase (MAPK) pathway. The binding of Ouabain to the Na^+^/K^+^ pump has been shown to activate Src kinase, which activates the Ras-Raf-MEK-ERK signaling cascade, a key component of the MAPK pathway^[11]^. This pathway plays a central role in cancer progression, as its dysregulation enhances tumor growth, survival, and resistance to apoptosis. Elevated MAPK signaling due to Na^+^/K^+^ pump modulation has been implicated in various malignancies, including breast, lung, and prostate cancer. Therapeutic strategies targeting this pathway, such as MEK inhibitors, have shown promise in preclinical and clinical studies^[12,13]^. Another important signaling pathway modulated by Ouabain is the phosphoinositide 3-kinase (PI3K) pathway, which plays a critical role in cell survival and metabolism. Ouabain binding to the Na^+^/K^+^ pump has been shown to activate PI3K, leading to the phosphorylation and activation of downstream effectors such as Akt and mTOR^[14]^. The PI3K-Akt-mTOR pathway is one of the most frequently altered pathways in cancer, with mutations leading to uncontrolled cell proliferation and survival. Inhibition of PI3K/Akt/mTOR signaling is a current therapeutic focus, particularly in hormone-driven cancers such as breast and prostate cancer, where targeting this pathway can enhance the efficacy of existing chemotherapies and immunotherapies^[15]^. In addition to the MAPK and PI3K pathways, Ouabain binding to the Na^+^/K^+^ pump has been shown to generate reactive oxygen species (ROS) by activating NADPH oxidase. The production of ROS is a critical component of cellular signaling, influencing processes such as apoptosis, inflammation, and cellular differentiation^[17]^. However, excessive ROS production contributes to oxidative stress, which is a key driver of pathologies such as hypertension, atherosclerosis, and neurodegenerative disorders^[18]^. In hypertension, elevated ROS levels disrupt endothelial function by reducing nitric oxide bioavailability, leading to vasoconstriction and increased vascular resistance. This oxidative stress-induced endothelial dysfunction is a major contributor to cardiovascular diseases. Antioxidant therapies that reduce ROS levels are currently being explored as potential treatments for hypertension and related disorders^[19]^. The interaction between Ouabain and the Na^+^/K^+^ pump also has significant implications for regulating intracellular calcium levels. The increase in intracellular sodium concentration resulting from Na^+^/K^+^ pump inhibition leads to the activation of the Na^+^/ Ca^2+^ exchanger, which excites sodium from the cell in exchange for calcium^[20]^. This exchange process can increase intracellular calcium levels, which are crucial in various cellular functions, including muscle contraction, neurotransmitter release, and gene expression^[22]^. Dysregulation of calcium homeostasis due to prolonged Na^+^/K^+^ pump inhibition has been linked to pathological cardiac hypertrophy and arrhythmias. Elevated intracellular calcium levels contribute to maladaptive cardiac remodeling, increasing the risk of heart failure. Targeting the Na^+^/K^+^ pump-mediated calcium flux may offer new therapeutic avenues for preventing hypertrophy-induced cardiac dysfunction^[24]^. The role of the Na^+^/K^+^ pump in cellular signaling extends beyond its interaction with Ouabain. The pump itself has been shown to interact with various proteins and signaling molecules, forming complexes that modulate its activity and function. One such interaction involves the caveolae, small invaginations in the plasma membrane that are rich in signaling molecules such as caveolin-1. The Na^+^/K^+^ pump has been found to associate with caveolae, and this interaction is thought to play a role in the spatial organization of signaling pathways within the cell^[25]^. Given these mechanistic insights, targeting the Na^+^/K^+^ pump and its downstream pathways is emerging as a novel strategy in disease treatment. Recent studies suggest that selective Na^+^/K^+^ pump modulators, such as cardiotonic steroids and small-molecule inhibitors, could be repurposed to treat cancer, hypertension, and neurodegenerative disorders. By refining these therapeutic interventions, researchers aim to harness the Na^+^/K^+^ pump’s signaling properties while minimizing adverse effects on its primary ion transport function^[5,23]^. The specific isoform composition of the Na^+^/K^+^ pump in a given tissue can influence its response to Ouabain and its role in cellular signaling. For example, the α2-isoform of the Na^+^/K^+^ pump, which is predominantly expressed in cardiac and skeletal muscle, has been shown to have a higher affinity for Ouabain compared to the α1-isoform, which is ubiquitously expressed^[28]^. This isoform-specific sensitivity to Ouabain has important implications for regulating ion homeostasis and signaling pathways in different tissues^[26]^. Moreover, the expression of specific Na^+^/K^+^ pump isoforms has been linked to various disease states, including hypertension, heart failure, and cancer, suggesting that the isoform composition of the pump may be a critical determinant of its function in health and disease^[27]^ Fig. 5 highlights the structural differences among the four human α isoforms, with homology models based on the potassium-occluded 3 KDP structure. The non-conservative substitutions shown help explain differences in Ouabain binding and isoform-specific regulation. The physiological impact of these isoform differences is illustrated in Table 3, which shows the significantly lower baseline mean blood pressure (MBP) in Ouabain-resistant α2 (α1R/Rα2R/R) mice compared to wild-type controls (α1R/Rα2S/S), measured by telemetry. In addition to its role in signaling pathways, the Na^+^/K^+^ pump has been implicated in regulating cellular energy metabolism. The pump’s activity is a major consumer of ATP in the cell, and its regulation is closely linked to the cell’s metabolic state^[6]^. Ouabain binding to the Na^+^/K^+^ pump has been shown to influence cellular metabolism by modulating the activity of metabolic enzymes and pathways. For example, Ouabain has been found to inhibit glycolysis by downregulating the activity of key glycolytic enzymes, leading to a shift in cellular energy production from glycolysis to oxidative phosphorylation^[8]^. This shift in metabolism can significantly affect cellular function, particularly in tissues with high energy demands, such as the heart and brain^[9]^. The regulation of cellular metabolism by the Na^+^/K^+^ pump and Ouabain underscores the pump’s role as a key integrator of cellular function and highlights its potential as a target for therapeutic intervention in metabolic diseases^[10]^. The involvement of the Na^+^/K^+^ pump in regulating immune responses represents another important aspect of its mechanistic pathways. Recent studies have shown that the Na^+^/K^+^ pump plays a role in the activation of immune cells and the modulation of inflammatory responses^[11]^. Ouabain binding to the Na^+^/K^+^ pump has been found to influence the activity of immune cells such as macrophages and T cells, producing pro-inflammatory cytokines and activating signaling pathways involved in immune responses^[12]^. The pump’s ability to modulate immune responses through its interaction with Ouabain highlights its role in the broader context of cellular signaling and function. The impact of the Na^+^/K^+^ pump on immune cell activity is particularly relevant in chronic inflammation, where dysregulation of the pump’s activity can contribute to the pathogenesis of autoimmune diseases, atherosclerosis, and other inflammatory conditions^[13]^. The modulation of immune responses by the Na^+^/K^+^ pump suggests that it may serve as a potential therapeutic target for treating inflammatory and immune-related disorders. The cross-talk between the Na^+^/K^+^ pump and other cellular signaling pathways further underscores the complexity of its mechanistic roles. For instance, interactions between the Na^+^/K^+^ pump and the renin-angiotensin-aldosterone system (RAAS) have been explored in the context of hypertension and cardiovascular diseases^[21]^. Ouabain has been found to increase the expression of angiotensin II receptors on vascular smooth muscle cells, thereby enhancing the vasoconstrictive effects of angiotensin II and contributing to the development of hypertension^[16]^. Additionally, the interaction between the Na^+^/K^+^ pump and RAAS components has been implicated in regulating sodium balance and fluid homeostasis, with potential implications for treating hypertension and related cardiovascular conditions^[29]^. The involvement of the Na^+^/K^+^ pump in regulating apoptosis represents another critical aspect of its mechanistic pathways. Apoptosis, or programmed cell death, is a tightly regulated process essential for maintaining tissue homeostasis and eliminating damaged or unwanted cells^[30]^. The Na^+^/K^+^ pump has been shown to regulate apoptosis through its effects on intracellular ion homeostasis and the activation of signaling pathways^[31]^. Ouabain binding to the Na^+^/K^+^ pump has been found to induce apoptosis in various cell types, including cancer cells, by disrupting the balance of intracellular ions and activating pro-apoptotic signaling pathways such as the MAPK and PI3K pathways^[32]^. The pro-apoptotic effects of Ouabain have been explored as a potential therapeutic strategy for cancer treatment, where the selective induction of apoptosis in cancer cells could lead to the inhibition of tumor growth and metastasis^[33]^. The regulation of cellular adhesion and motility by the Na^+^/K^+^ pump is another important mechanistic pathway explored in recent years. Cellular adhesion and motility are critical processes involved in tissue development, wound healing, and the metastatic spread of cancer cells^[34]^. The Na^+^/K^+^ pump has been found to interact with various components of the cytoskeleton and cell adhesion molecules, influencing the organization of the cytoskeleton and the formation of cell-matrix adhesions^[35]^. Ouabain binding to the Na^+^/K^+^ pump has been shown to modulate these processes by altering the activity of signaling pathways involved in cytoskeletal dynamics and cell adhesion, such as the RhoA/ROCK and Rac1 pathways^[36]^. The ability of Ouabain to influence cellular adhesion and motility has potential implications for treating diseases characterized by abnormal cell migration, such as cancer and chronic inflammatory conditions^[37]^.

**Table 3:** Baseline MBP in awake WT (α1 ^R/R^α2^S/S^) mice and Ouabain-resistant α2 (α1 ^R/R^α2 ^R/R^) mice measured by telemetry; ΔMBP is the difference between the two

Study	α1 ^R/R^α2^S/S^(WT) MBP, mm Hg	α1 ^R/R^α2 ^R/R^MBP, mm Hg	ΔMBP, mm Hg
1.^a^	104.0 ± 2.6 (11)^b^	108.2 ± 3.2 (10)	4.2
2.^c^	115 ± 3 (8)	125 ± 5 (10)	10
3a.^d^	121 ± 8 (4)	128 ± 3 (5)	7
3b.^d^	115 ± 6 (5)	125 ± 4 (7)	10
4.^d^	97.8 ± 0.8 (7)	108.2 ± 0.8 (7)	10.4

^a^Data from unpublished study (personal communication or in-house data).

^b^Number in parentheses indicates number of animals used.

^c^Data extracted from previously published study.

^d^Data from multiple sub-studies within the same experimental framework or study series.

Values are presented as mean ± SE. Mice were matched by age, weight, and sex. Numbers in parentheses indicate sample sizes. Mouse sex was unspecified in some cases. For Experiment 3, part a and b represent separate trials using male mice. WT = wild type; MBP = mean blood pressure (adapted from Blaustein, 2018).

## Evolution of the concept over four decades

**Figure 6.**
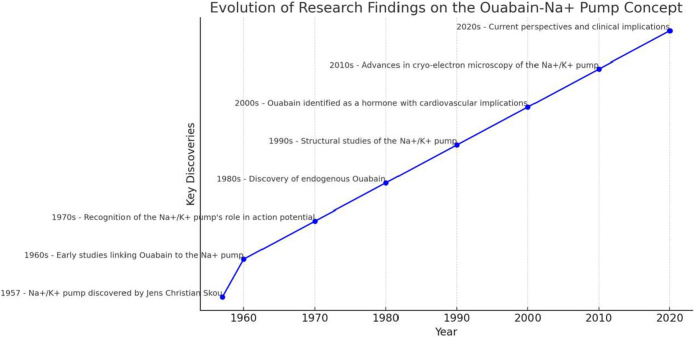
Historical trends in Ouabain-Na^+^ pump research. This graph can visually represent the evolution of research findings, highlighting fundamental discoveries and shifts in understanding related to the Ouabain-Na^+^ pump concept. Source: authors’ creations.

The past decade has seen a growing interest in the potential therapeutic applications targeting the Na^+^/K^+^ pump. The concept of the pump as a key player in an endocrine-like regulatory system has led to exploring new drug candidates that modulate its activity. For example, researchers have investigated using Na^+^/K^+^ pump inhibitors, such as cardiac glycosides, to treat cancer. It has been found that these compounds can induce apoptosis in cancer cells by disrupting ion homeostasis and activating cell death pathways, making the Na^+^/K^+^ pump an attractive target for anticancer therapies^[26-28]^. In addition to cardiac glycosides, novel small-molecule inhibitors and monoclonal antibodies targeting specific isoforms of the Na^+^/K^+^ pump are being developed to enhance selectivity and minimize off-target effects. Preclinical studies have demonstrated promising anticancer effects, particularly in glioblastoma and pancreatic cancer models, where Na^+^/K^+^ pump modulation alters tumor cell metabolism and immune evasion mechanisms. Clinical trials are currently underway to evaluate the efficacy of these inhibitors in various types of cancer, including breast, prostate, and lung cancer. In parallel, efforts have been made to develop drugs that selectively modulate the Na^+^/K^+^ pump’s signaling functions without affecting its ion transport activity. Such compounds could potentially offer therapeutic benefits for conditions like hypertension and heart failure, where excessive Na⁺/K⁺ pump inhibition by endogenous Ouabain contributes to disease progression. One notable example is rostafuroxin (PST2238), a digitoxigenin derivative that selectively antagonizes endogenous Ouabain^[6,8]^. Phase II clinical trials (e.g., OSEPH, NCT00415038) have demonstrated that rostafuroxin can effectively lower blood pressure in patients with salt-sensitive hypertension, particularly those carrying specific α-adducin polymorphisms. Another compound, Digibind (anti-digoxin antibody), has shown potential in neutralizing endogenous cardiotonic steroids in preclinical models of heart failure. Though primarily used in digoxin toxicity, studies have explored its broader cardiovascular applications. These findings highlight that modulating Na⁺/K⁺ pump signaling – rather than outright inhibition – may offer safer, more targeted cardiovascular therapy^[9,10]^. Fig. 6 illustrates this evolution, providing a visual summary of the key milestones, therapeutic advances, and paradigm shifts that have shaped our understanding of the Na⁺/K⁺ pump as an endocrine modulator.

The evolution of the Na^+^/K^+^ pump concept over the past four decades has also been marked by advances in our understanding of its structure and molecular dynamics. High-resolution structural studies, including X-ray crystallography and cryo-electron microscopy, have provided detailed insights into the pump’s conformational changes during the transport cycle and its interaction with ligands like Ouabain^[12,13,21]^. These structural insights have also guided rational drug design efforts to improve the specificity of Na^+^/K^+^ pump-targeting agents. In particular, computational docking and AI-driven molecular modeling techniques are used to identify novel binding pockets that could serve as drug targets, expanding the repertoire of potential Na^+^/K^+^ pump modulators beyond traditional cardiac glycosides. Looking forward, the evolution of the Na^+^/K^+^ pump concept will likely be driven by advancements in technologies such as single-cell transcriptomics, proteomics, and systems biology. These approaches will enable researchers to map the Na^+^/K^+^ pump’s activity and regulation in unprecedented detail, revealing how it functions in different cell types and tissues under various physiological and pathological conditions. Additionally, the integration of these data into computational models of cellular and systemic physiology will help to refine our understanding of the pump’s role in health and disease, paving the way for new diagnostic and therapeutic strategies^[13,19,20]^.

## The pump, the exchanger, and the Holy Spirit

**Figure 7.**
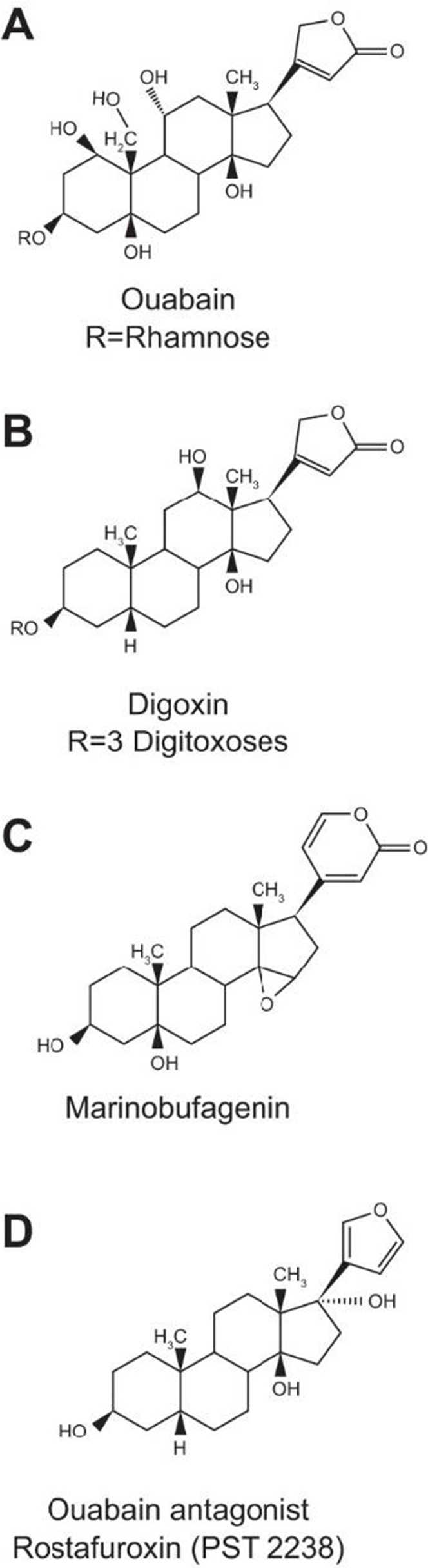
Structural comparison of cardiotonic steroids involved in Na⁺/K⁺ pump modulation. Structures of Ouabain, digoxin, marinobufagenin, and rostafuroxin are shown. The sugar components of Ouabain and digoxin are labeled as “Rs” – source: Blaustein MP. The pump, the exchanger, and the holy spirit: origins and 40-year evolution of ideas about the Ouabain-Na^+^ pump endocrine system. Am J Physiol Cell Physiol. 2018 Jan 1;314(1):C3-C26. Doi: 10.1152/ajpcell.00196.2017.

The Na^+^/K^+^ pump, also known as the sodium-potassium ATPase, was first described by Jens Christian Skou in 1957, earning him the Nobel Prize in Chemistry in 1997. The pump’s primary function is to maintain the electrochemical gradients of sodium (Na^+^) and potassium (K^+^) ions across the plasma membrane by actively transporting three Na^+^ ions out of the cell and two K^+^ ions into the cell against their concentration gradients, using energy derived from ATP hydrolysis^[1-3]^. This action is essential for various physiological processes, including maintaining membrane potential, regulating cell volume, and providing the driving force for secondary active transport systems. Fig. 7 illustrates the structure of the Na⁺/K⁺ pump and its interaction with endogenous Ouabain, highlighting its dual role in ion transport and signal transduction. Beyond these traditional roles, the Na^+^/K^+^ pump has been increasingly recognized for its involvement in signal transduction pathways. The binding of endogenous cardiac glycosides, such as Ouabain, to the Na^+^/K^+^ pump has been shown to activate a cascade of intracellular signaling events, including the Src kinase and MAPK/ERK pathways, which can influence cell proliferation, apoptosis, and differentiation^[4-6]^. This signaling function positions the Na^+^/K^+^ pump as a key player in cellular communication and regulation, extending its impact beyond ion homeostasis. The concept of the Na^+^/K^+^ pump as part of an endocrine-like system has gained considerable traction. Endogenous Ouabain and related compounds are now considered hormones, influencing various physiological processes, particularly in the cardiovascular system. Elevated levels of endogenous Ouabain have been associated with hypertension, heart failure, and other cardiovascular diseases, suggesting that the Na^+^/K^+^ pump may play a role in the long-term regulation of blood pressure and fluid balance^[7-9]^. This regulatory function has prompted a reevaluation of the Na^+^/K^+^ pump’s role in physiology, positioning it as a central component of an intricate signaling network. Ion exchangers, particularly the Na^+^/ Ca^2+^ exchanger (NCX), are closely intertwined with the function of the Na^+^/K^+^ pump. The NCX operates in a bidirectional manner, allowing the exchange of three Na^+^ ions for one Ca^2+^ ion, depending on the electrochemical gradients of these ions across the plasma membrane. The activity of the Na^+^/K^+^ pump is crucial for maintaining the Na^+^ gradient that drives the NCX, thereby indirectly regulating intracellular calcium levels^[10-12]^. Calcium ions are vital second messengers involved in numerous cellular processes, including muscle contraction, neurotransmitter release, and gene expression. This relationship is visually depicted in Fig. 7, where the functional coupling between the Na⁺/K⁺ pump and the NCX is represented as a dynamic regulatory loop. The interplay between the Na^+^/K^+^ pump and the NCX exemplifies how ion transporters and exchangers work in concert to regulate cellular homeostasis and signaling. The relationship between the Na^+^/K^+^ pump and the NCX is particularly significant in cardiac physiology. The precise regulation of intracellular calcium levels is essential for proper cardiac function, as calcium plays a central role in the excitation-contraction coupling of cardiac myocytes. The Na^+^/K^+^ pump, by maintaining the Na^+^ gradient, indirectly controls the activity of the NCX and, thus, the intracellular calcium concentration^[5]^. Dysregulation of this system can lead to pathological conditions such as arrhythmias and heart failure. For example, an increased intracellular Na^+^ concentration due to impaired Na^+^/K^+^ pump activity can reduce the driving force for the NCX, leading to elevated intracellular Ca^2+^ levels and increased cardiac contractility. While this may initially enhance cardiac output, chronic elevation of intracellular Ca^2+^ can lead to maladaptive remodeling and heart failure^[6]^. In addition to its role in the cardiovascular system, the interaction between the Na^+^/K^+^ pump and ion exchangers like the NCX has implications for other physiological systems, including the nervous system and the kidneys. In neurons, the Na^+^/K^+^ pump and NCX collaborate to regulate synaptic transmission and plasticity by controlling the intracellular concentrations of Na^+^ and Ca^2+^. Proper functioning of this system is essential for cognitive processes such as learning and memory^[7]^. In the kidneys, the Na^+^/K^+^ pump is involved in sodium reabsorption and regulating fluid balance, which is also influenced by various ion exchangers and channels^[8]^. The coordination between the Na^+^/K^+^ pump and these exchangers is crucial for maintaining systemic homeostasis, and disruptions in this balance can contribute to conditions such as hypertension and chronic kidney disease. The metaphorical reference to the “Holy Spirit” in the context of the Na^+^/K^+^ pump and ion exchangers invites a deeper philosophical reflection on the nature of this regulatory system. The term “Holy Spirit” has traditionally been used in religious contexts to denote a guiding or animating force, often associated with wisdom, inspiration, and the sustenance of life. In the scientific discourse surrounding the Na^+^/K^+^ pump, this metaphor can be interpreted as recognizing the pump’s central role in maintaining the delicate balance of life at the cellular level. Just as the Holy Spirit is believed to be an unseen force that guides and sustains life, the Na^+^/K^+^ pump operates behind the scenes, orchestrating the ion gradients that are essential for cellular function and survival^[9,13,21]^. This metaphor also underscores the interconnectedness of the Na^+^/K^+^ pump with other components of the cellular machinery. The pump does not operate in isolation but is part of a larger network of ion transporters, exchangers, and signaling molecules that work together to regulate cellular homeostasis. The reference to the “Holy Spirit” can be seen as a symbolic acknowledgment of the pump’s integrative role within this network, much like the way the Holy Spirit is often conceived as a unifying presence in religious thought^[10,14,15]^. Furthermore, the “Holy Spirit” metaphor can be extended to highlight the Na^+^/K^+^ pump’s involvement in transmitting signals and regulating cellular communication. In this context, the pump can be viewed as a mediator that facilitates the flow of information within and between cells, similar to how the Holy Spirit is believed to facilitate communication and understanding in religious contexts^[11,16,17]^. This perspective emphasizes the pump’s role in ensuring that cellular signals are properly transmitted and interpreted, a critical function for coordinating complex physiological processes. The evolution of the Na^+^/K^+^ pump concept over the past several decades has been marked by significant advances in understanding its structure, function, and regulation. High-resolution structural studies have provided detailed insights into the pump’s conformational changes during the transport cycle and its interaction with ligands such as Ouabain^[12,18,19]^. These studies have not only deepened our understanding of the pump’s mechanism of action but have also informed the development of new therapeutic strategies targeting the pump in various diseases. For example, cardiac glycosides that inhibit the Na^+^/K^+^ pump are currently being explored as potential treatments for cancer, as they can induce apoptosis in cancer cells by disrupting ion homeostasis^[13]^. In addition to structural studies, molecular biology, and biochemistry advancements have shed light on the regulatory mechanisms that modulate Na^+^/K^+^ pump activity. Post-translational modifications, such as phosphorylation and ubiquitination, have been shown to influence the pump’s activity and stability, providing additional layers of control over its function^[20-22]^. These regulatory mechanisms are crucial for the pump’s ability to respond to cellular environment changes and participate in signaling pathways that influence cell behavior. The Na^+^/K^+^ pump’s role in disease has also become a major research focus, particularly cardiovascular and neurological disorders. Elevated levels of endogenous Ouabain and other cardiac glycosides have been implicated in the pathogenesis of hypertension and heart failure, conditions that are characterized by dysregulation of sodium and fluid balance^[14]^. Therapeutic interventions targeting the Na^+^/K^+^ pump are being developed to restore normal pump function and alleviate these conditions. For example, inhibitors of the pump’s signaling functions are being investigated as potential treatments for hypertension. In contrast, drugs that modulate the pump’s activity are being explored for their neuroprotective effects in conditions such as stroke and neurodegenerative diseases^[15]^. The “Holy Spirit” metaphor can also be applied to the therapeutic potential of targeting the Na^+^/K^+^ pump. Just as the Holy Spirit is often invoked as a source of healing and restoration in religious contexts, the Na^+^/K^+^ pump can be seen as a target for therapeutic interventions that aim to restore cellular homeostasis and promote healing in various diseases. This perspective highlights the pump’s central role in maintaining the balance of life at the cellular level. It underscores the importance of developing therapies that can modulate its activity in a precise and targeted manner^[16,24,25]^. The interplay between the Na^+^/K^+^ pump and ion exchangers such as the NCX has further expanded our understanding of the pump’s role in health and disease. The NCX plays a crucial role in muscle contraction, neurotransmitter release, and gene expression by regulating intracellular calcium levels. The Na^+^/K^+^ pump, by maintaining the Na^+^ gradient that drives the NCX, indirectly influences these processes and contributes to the regulation of cellular homeostasis^[17]^. Dysregulation of this interplay can lead to pathological conditions, as exemplified by the role of the Na^+^/K^+^ pump and NCX in the development of cardiac arrhythmias and heart failure^[18]^. The relationship between the Na^+^/K^+^ pump and ion exchangers also has implications for developing new therapeutic strategies. Drugs that target the Na^+^/K^+^ pump or the NCX could modulate this interplay, offering potential treatments for conditions such as heart failure, arrhythmias, and hypertension. For instance, specific inhibitors or modulators of the Na^+^/K^+^ pump could be designed to fine-tune its activity, thereby adjusting the Na^+^ gradient to optimize NCX function. This approach could help restore the balance of calcium homeostasis in cardiac cells, preventing the maladaptive remodeling associated with chronic heart failure and improving overall cardiac function^[10^-,^12]^. The intricate relationship between the Na^+^/K^+^ pump and NCX is also relevant in neurodegenerative diseases. Proper calcium signaling is essential for neurotransmission and synaptic plasticity in neurons, which underlie learning and memory. Dysregulation of calcium homeostasis, often linked to impaired Na^+^/K^+^ pump function, has been implicated in conditions such as Alzheimer’s disease, Parkinson’s disease, and amyotrophic lateral sclerosis (ALS)^[12,13,19]^. Therapeutic strategies targeting neurons’ Na^+^/K^+^ pump or NCX could mitigate the excessive calcium influx contributing to neuronal damage and degeneration in these disorders. The metaphorical use of the “Holy Spirit” in the context of the Na^+^/K^+^ pump invites a broader philosophical exploration of the pump’s role in sustaining life at the cellular level. In many religious traditions, the Holy Spirit is perceived as an invisible force that breathes life into beings, offering guidance, protection, and healing. Similarly, the Na^+^/K^+^ pump, though unseen and operating silently within the cell, is vital for maintaining the conditions necessary for life. By establishing and regulating the electrochemical gradients of sodium and potassium, the pump ensures that cells can perform their essential functions, from generating action potentials in neurons to contracting muscles and maintaining osmotic balance^[13-15]^. In this analogy, the Holy Spirit represents the life-giving force of the Na^+^/K^+^ pump and its capacity to mediate communication and coordination within and between cells. Just as the Holy Spirit is often described as facilitating communication between the divine and humanity, the Na^+^/K^+^ pump plays a crucial role in facilitating cellular communication through its influence on signaling pathways. By regulating ion gradients, the pump controls the excitability of neurons, the contraction of muscles, and the release of hormones, all of which are critical for the coordination of complex physiological processes^[4,16,17]^. The pump’s role in these processes is further underscored by its involvement in regulating systemic homeostasis. The Na^+^/K^+^ pump is essential for sodium reabsorption in the kidneys, a process crucial for maintaining blood pressure and fluid balance. In the cardiovascular system, the pump’s activity influences vascular tone and cardiac output, thereby contributing to the long-term regulation of blood pressure. Dysregulation of the Na^+^/K^+^ pump, whether due to genetic mutations, acquired conditions, or external factors such as drugs, can lead to significant disturbances in these homeostatic mechanisms, resulting in hypertension, heart failure, and other cardiovascular diseases^[5,18,19]^. Integrating the Na^+^/K^+^ pump into the broader endocrine-like system further emphasizes its role as a mediator of communication and regulation in the body. Endogenous cardiac glycosides, such as Ouabain, bind to the Na^+^/K^+^ pump and modulate its activity, influencing various physiological processes. These glycosides are now recognized as hormones regulating blood pressure, fluid balance, and cellular growth and differentiation. The concept of the Na^+^/K^+^ pump as part of an endocrine system underscores its central role in maintaining the balance of life at the cellular and systemic levels^[6-8]^. The metaphor of the Holy Spirit can also be extended to consider the Na^+^/K^+^ pump’s role in disease and healing. In conditions where the pump’s function is impaired, such as in certain forms of heart failure or neurodegenerative diseases, restoring the pump’s activity can be seen as healing. Therapeutic interventions targeting the Na^+^/K^+^ pump, whether through drugs that enhance its activity or gene therapy to correct genetic mutations, can potentially restore cellular function and improve patient outcomes. In this sense, the Na^+^/K^+^ pump can be viewed as a vital component of the body’s innate ability to heal and maintain homeostasis^[20,22,24]^. The ongoing research into the Na^+^/K^+^ pump continues to uncover new aspects of its function and regulation, shedding light on its role in health and disease. Advances in structural biology have provided detailed insights into the pump’s conformational changes during its transport cycle, offering new opportunities to develop targeted therapies. For example, high-resolution crystallography and cryo-electron microscopy have revealed the precise binding sites for cardiac glycosides on the Na^+^/K^+^ pump, paving the way for the design of novel drugs that can modulate the pump’s activity with greater specificity and fewer side effects^[5,8,25]^. In addition to its structural role, the Na^+^/K^+^ pump’s involvement in signal transduction pathways has opened up new avenues for research into its role in cellular communication and disease. The pump’s ability to activate signaling cascades, such as the Src kinase and MAPK/ERK pathways, positions it as a key regulator of cell growth, differentiation, and apoptosis. Dysregulation of these pathways has been implicated in a variety of diseases, including cancer, where the Na^+^/K^+^ pump’s role as a signaling hub could be targeted for therapeutic intervention^[9,23,28]^. Exploring the Na^+^/K^+^ pump’s role in disease has also extended to its potential as a biomarker for various conditions. For example, elevated levels of endogenous Ouabain have been proposed as a biomarker for hypertension and heart failure, reflecting the pump’s involvement in the pathophysiology of these conditions. Similarly, alterations in the expression or activity of the Na^+^/K^+^ pump have been observed in certain types of cancer, suggesting that the pump could serve as a diagnostic or prognostic marker in these diseases^[10-12]^. The Holy Spirit metaphor can also be considered in the context of the Na^+^/K^+^ pump’s adaptability and resilience in the face of stress. Just as the Holy Spirit is often invoked as a source of strength and guidance in times of adversity, the Na^+^/K^+^ pump is essential for maintaining cellular function under stress conditions, such as hypoxia, ischemia, or oxidative damage. The pump’s ability to rapidly respond to changes in the cellular environment by adjusting its activity to meet the demands of the cell underscores its role as a stabilizing and protective force within the cell^[11,13,21]^. The therapeutic implications of targeting the Na^+^/K^+^ pump are vast and continue to be explored across various diseases. In cardiovascular diseases, drugs that modulate the pump’s activity are being developed to treat conditions such as heart failure, arrhythmias, and hypertension. In cancer, the pump is being investigated as a target for therapies that can disrupt the ion homeostasis of cancer cells, leading to their selective apoptosis. In neurological disorders, strategies to enhance the pump’s activity are being explored as potential treatments for conditions characterized by impaired synaptic transmission and neuronal survival^[12,14,15]^. Integrating the Na^+^/K^+^ pump into a broader conceptual framework that includes ion exchangers, signaling pathways, and even metaphorical interpretations such as the Holy Spirit highlights the pump’s multifaceted role in biology. Far from being a simple ion transporter, the Na^+^/K^+^ pump is a dynamic and adaptable regulator of cellular function, capable of influencing a wide range of physiological processes and responding to the challenges of disease. As research continues to uncover new dimensions of the pump’s activity, its importance in maintaining the balance of life at both the cellular and systemic levels becomes increasingly apparent^[13,16,17]^.

## Endocrine regulation

**Figure 8.**
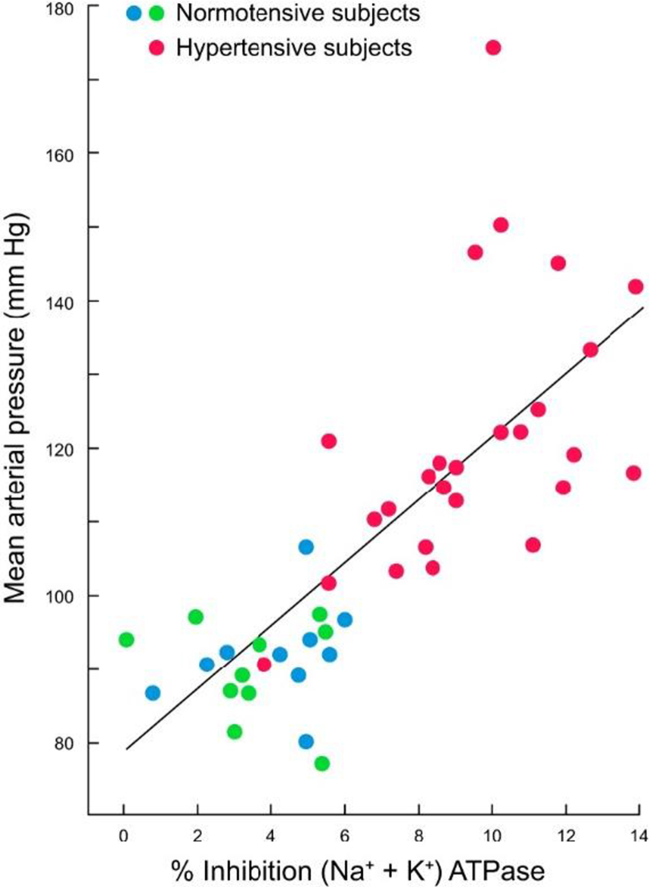
Strong positive correlation between plasma Na⁺/K⁺-ATPase inhibition and mean arterial pressure in normotensive and hypertensive individuals. Mean arterial pressure (MAP) strongly correlates with plasma Na⁺/K⁺-ATPase (NKA) inhibitory activity. The data include 20 normotensive and 26 hypertensive individuals, with samples collected as single draws (green) or integrated over 6 hours (red and blue). A significant linear relationship was observed (*r* = 0.73, *P* < 0.0005), suggesting a link between elevated MAP and increased NKA inhibition. Source: Blaustein^[1]^.

**Figure 9.**
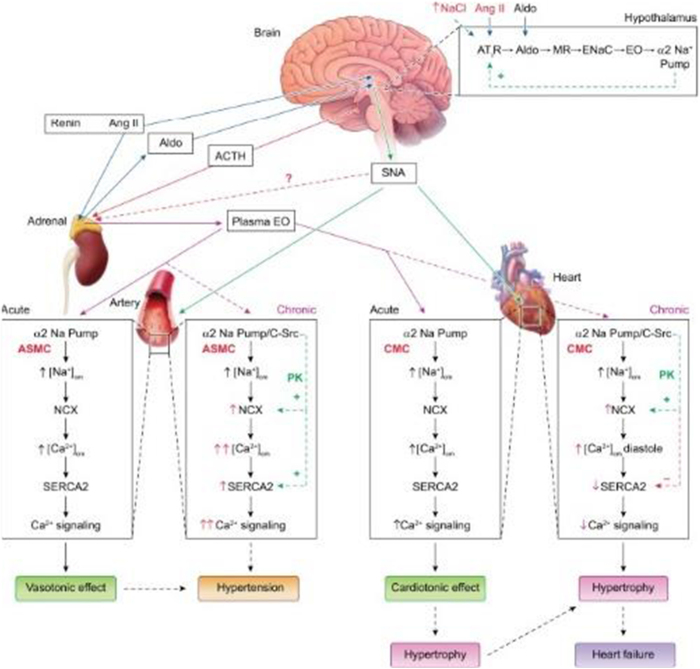
Regulation of heart and vascular function via sympathetic and neurohumoral pathways in hypertension and heart failure. This diagram outlines how centrally controlled sympathetic and neurohumoral pathways regulate heart and vessel function, contributing to hypertension and heart failure. Angiotensin II and high salt levels activate hypothalamic AT1 receptors, increasing sympathetic output. This triggers vasoconstriction and stronger cardiac contraction. A separate hypothalamic pathway involving aldosterone, ENaC, endogenous Ouabain, and α2 Na^+^ pumps amplifies sympathetic activity and EO release. EO inhibits α2 pumps, raising intracellular Na^+^ and Ca^2+^, enhancing vascular and cardiac tone. Chronically elevated EO also activates kinase pathways that increase NCX and SERCA2 expression, further promoting calcium signaling. In the heart, impaired Na^+^ gradients and altered SERCA2 function disrupt relaxation and contraction, contributing to heart failure. Source: Blaustein MP. The pump, the exchanger, and the holy spirit: origins and 40-year evolution of ideas about the Ouabain-Na^+^ pump endocrine system. Am J Physiol Cell Physiol. 2018 Jan 1;314(1):C3-C26. Doi: 10.1152/ajpcell.00196.2017.

**Figure 10.**
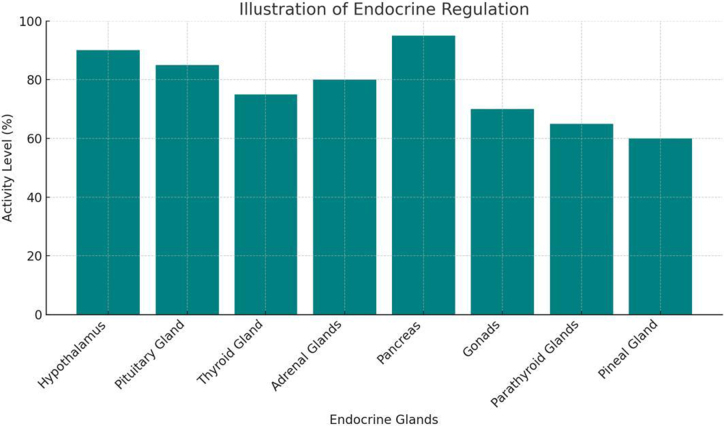
Relative activity levels of endocrine glands in endocrine regulation. Here is a bar graph illustrating the activity levels of various endocrine glands as part of the overall endocrine regulation. Each gland plays a crucial role in maintaining physiological balance, with varying degrees of activity. This graph serves as a conceptual representation of their contribution to endocrine regulation. Source: Authors’ creations.

Hormones are signaling molecules that interact with specific receptors on target cells to trigger a biological response. They can be classified into several categories based on their chemical structure, including peptides, steroids, and amines. Peptide hormones, such as insulin and growth hormone, are composed of amino acids and are usually synthesized as precursor molecules that require cleavage to become active. Steroid hormones, like cortisol and estrogen, are derived from cholesterol and are lipid-soluble, allowing them to diffuse through cell membranes to bind to intracellular receptors. Amines, such as thyroxine and epinephrine, are derived from amino acids and can act on both cell surface and intracellular receptors^[10,12,13]^. The regulation of hormone levels is crucial for maintaining physiological balance. Feedback loops typically control hormone secretion, which can be either negative or positive. Negative feedback loops are the most common, maintaining hormone levels within a narrow range. In a negative feedback loop, an increase in the level of a particular hormone inhibits further secretion of that hormone, preventing excessive accumulation. For example, the hypothalamic-pituitary-adrenal (HPA) axis operates through a negative feedback mechanism, where elevated cortisol levels inhibit the release of corticotropin-releasing hormone (CRH) from the hypothalamus and adrenocorticotropic hormone (ACTH) from the pituitary gland, thereby reducing cortisol production by the adrenal cortex^[20,22]^. In contrast, positive feedback loops amplify the response to a stimulus, leading to a greater hormone release. This type of feedback is less common but plays a crucial role in certain physiological processes. An example of a positive feedback loop is the luteinizing hormone (LH) surge that triggers ovulation. Here, rising estrogen levels stimulate the anterior pituitary gland to release a large amount of LH, which in turn leads to the release of the egg from the ovary^[13,21]^. Endocrine regulation also involves complex interactions between hormones, which can act synergistically, antagonistically, or permissively. Synergistic effects occur when two or more hormones work together to produce a greater effect than each other. For instance, the combined action of glucagon and epinephrine enhances glucose release from the liver more effectively than either hormone would individually. Antagonistic effects occur when one hormone opposes the action of another. Insulin and glucagon provide a classic example, where insulin lowers blood glucose levels while glucagon raises them. Permissive effects occur when one hormone’s presence enhances another’s action. Thyroid hormones, for example, enhance the effects of catecholamines on the heart by increasing the number of beta-adrenergic receptors^[14,15]^. A simplified visual overview of these hormonal interactions – including feedback mechanisms, hormone-receptor relationships, and systemic integration – is presented in Fig. 8. Additionally, the multidimensional influence of endocrine regulation across immune, cardiovascular, and metabolic systems is further illustrated in Fig. 9. A thorough illustration of the endocrine system’s integration and coordination with organ-specific axes – such as the HPA, HPT, and HPG axes – is provided in Fig. 10. Fig. 10 demonstrates the hierarchical control exerted by the hypothalamus and pituitary gland and highlights major hormonal feedback loops and effector responses. These figures (8, 9, and 10) illustrate how endocrine signals regulate diverse physiological functions through dynamic and reciprocal interactions across organ systems. The endocrine system is intricately linked with the nervous system, forming the neuroendocrine system. The hypothalamus, a brain region, plays a pivotal role in this system by bridging the nervous and endocrine systems. It releases and inhibits hormones that control pituitary hormone secretion, regulating various endocrine glands. The hypothalamus receives input from other brain regions and the body, allowing it to integrate various signals and maintain homeostasis. This integration is crucial for the body’s response to stress, reproduction, and energy balance^[16,17]^. One of the key axes in endocrine regulation is the hypothalamic-pituitary-thyroid (HPT) axis, which regulates metabolism. The hypothalamus secretes thyrotropin-releasing hormone (TRH), which stimulates the anterior pituitary to release thyroid-stimulating hormone (TSH). TSH then stimulates the thyroid gland to produce and release thyroid hormones, primarily thyroxine (T4) and triiodothyronine (T3). These hormones regulate metabolic rate, heat production, and other critical functions. The levels of thyroid hormones are tightly regulated by a negative feedback loop, where high levels of T4 and T3 inhibit the release of TRH and TSH^[9,10]^. The hypothalamic-pituitary-gonadal (HPG) axis is another crucial component of endocrine regulation, particularly in the control of reproduction. The hypothalamus releases gonadotropin-releasing hormone (GnRH), which stimulates the anterior pituitary to secrete LH and follicle-stimulating hormone (FSH). These gonadotropins regulate the function of the ovaries and testes, including the production of sex hormones such as estrogen, progesterone, and testosterone. The HPG axis controls the menstrual cycle in females while it regulates spermatogenesis in males. The secretion of sex hormones is also regulated by negative feedback mechanisms, where elevated estrogen or testosterone levels inhibit the release of GnRH, LH, and FSH^[11,12]^. Endocrine regulation extends to maintaining calcium homeostasis, a process controlled by the parathyroid glands, thyroid gland, and kidneys. The parathyroid glands secrete parathyroid hormone (PTH) in response to low blood calcium levels. PTH increases calcium levels by stimulating calcium release from bones, increasing calcium reabsorption in the kidneys, and promoting the activation of vitamin D in the kidneys, which enhances calcium absorption in the intestines. Conversely, the thyroid gland secretes calcitonin, a hormone that lowers blood calcium levels by inhibiting bone resorption and increasing calcium excretion by the kidneys. The balance between PTH and calcitonin ensures that blood calcium levels remain within a narrow range, essential for proper nerve and muscle function^[13,21]^. Another critical aspect of endocrine regulation is the control of glucose homeostasis, primarily governed by insulin and glucagon hormones produced by the pancreas. Insulin is secreted in response to elevated blood glucose levels, such as after a meal. It promotes glucose uptake by cells, particularly in the liver, muscle, and adipose tissue. It also inhibits gluconeogenesis and glycogenolysis, processes that generate glucose. Glucagon, on the other hand, is released during fasting or low blood glucose levels and stimulates the liver to produce and release glucose through glycogenolysis and gluconeogenesis. The balance between insulin and glucagon maintains blood glucose levels within a narrow range, ensuring a steady supply of energy to the body^[14,15]^. The endocrine regulation of water and electrolyte balance is another vital function, primarily mediated by the hypothalamic-pituitary-adrenal (HPA) axis and the renin-angiotensin-aldosterone system (RAAS). The HPA axis is activated in response to stress, releasing CRH from the hypothalamus, which stimulates the pituitary to secrete ACTH. ACTH then prompts the adrenal cortex to produce cortisol, a glucocorticoid that regulates metabolism, immune response, and stress adaptation. Cortisol also has mineralocorticoid activity, influencing sodium and water retention by the kidneys^[5,6]^. The RAAS plays a crucial role in blood pressure regulation and fluid balance. When blood pressure drops, the kidneys release renin, an enzyme that converts angiotensinogen into angiotensin I. The lungs’ angiotensin-converting enzyme (ACE) then convert Angiotensin II to angiotensin II. Angiotensin II is a potent vasoconstrictor that increases blood pressure and stimulates the adrenal glands to release aldosterone. Aldosterone promotes sodium and water reabsorption in the kidneys, increasing blood pressure^[16,17]^. The endocrine system controls the regulation of energy balance and body weight, particularly through hormones like leptin, Ghrelin, and insulin. Leptin is produced by adipose tissue and acts on the hypothalamus to suppress appetite and increase energy expenditure. It provides feedback to the brain about the body’s energy stores, helping to maintain a stable body weight. Conversely, Ghrelin is secreted by the stomach and stimulates appetite by acting on the hypothalamus. Insulin, in addition to its role in glucose homeostasis, also affects appetite regulation and energy balance. Dysregulation of these hormones can lead to obesity, metabolic syndrome, and type 2 diabetes^[18,19]^. Endocrine regulation is also crucial in the stress response, involving multiple hormones and physiological systems. The HPA axis is central to the stress response, with the release of cortisol being a key event. Cortisol helps the body cope with stress by mobilizing energy stores, suppressing non-essential functions like digestion and reproduction, and modulating the immune response. The sympathetic nervous system, closely linked to the endocrine system, is also activated during stress, releasing catecholamines like epinephrine and norepinephrine from the adrenal medulla. These hormones prepare the body for a “fight or flight” response by increasing heart rate, blood pressure, and glucose availability^[20,22]^. In addition to these well-characterized roles, the endocrine system is involved in various other physiological processes, including growth and development, reproductive function, and immune regulation. Growth hormone (GH), produced by the anterior pituitary, is essential for normal growth and development in children. It stimulates the growth of bones and tissues by promoting protein synthesis and increasing cell proliferation. GH also has metabolic effects, including stimulating lipolysis and reducing tissue glucose uptake, thereby increasing blood glucose levels. The secretion of GH is regulated by a balance of stimulatory and inhibitory factors, with growth hormone-releasing hormone (GHRH) from the hypothalamus promoting its release and somatostatin inhibiting it. The pulsatile release of GH, which occurs in response to factors like sleep, exercise, and stress, is essential for its physiological actions. Insulin-like growth factors (IGFs), produced in response to GH, mediate many of its growth-promoting effects by stimulating cell proliferation and differentiation, particularly in bone and cartilage^[1,2]^. The endocrine system also tightly controls reproductive function through the coordinated actions of hormones from the hypothalamus, pituitary gland, and gonads. In females, the menstrual cycle is regulated by the cyclic release of hormones, including estrogen, progesterone, LH, and FSH. These hormones control the growth and maturation of ovarian follicles, ovulation, and endometrium preparation for potential pregnancy. In males, LH and FSH regulate spermatogenesis and testosterone production by the testes, which is essential for the development of male secondary sexual characteristics and reproductive function^[3,4]^. The immune system is another area where endocrine regulation plays a significant role. Hormones such as cortisol, adrenaline, and thyroid hormones influence immune function by modulating the activity of immune cells, cytokine production, and inflammatory responses. Cortisol, for example, has potent anti-inflammatory effects and is often used therapeutically to treat inflammatory and autoimmune diseases. However, chronic stress and prolonged exposure to high cortisol levels can suppress immune function and increase infection susceptibility^[5,6]^. Thyroid hormones, primarily T3 and T4, are also crucial for immune regulation. They influence the maturation and function of immune cells, including T lymphocytes and natural killer cells, and modulate the production of cytokines. Dysregulation of thyroid hormone levels, as seen in conditions like hypothyroidism or hyperthyroidism, can lead to altered immune function and increased risk of autoimmune diseases^[7,8]^. Through the secretion of melatonin, the pineal gland also plays a role in endocrine regulation, particularly in circadian rhythms and sleep-wake cycles. Melatonin is produced in response to darkness and helps regulate sleep timing by acting on receptors in the brain’s suprachiasmatic nucleus (SCN), the body’s master circadian clock. In addition to its role in sleep regulation, melatonin has antioxidant properties and modulates immune function, potentially influencing the body’s response to infections and cancer^[9,10]^. Another important aspect of endocrine regulation is the role of the endocrine pancreas in maintaining energy homeostasis. The pancreas contains clusters of cells known as the islets of Langerhans, which include alpha, beta, and delta cells. Beta cells produce insulin, lowering blood glucose levels by promoting glucose uptake and tissue storage. Alpha cells produce glucagon, which raises blood glucose levels by stimulating liver glycogen breakdown and glucose production. Delta cells produce somatostatin, inhibiting insulin and glucagon release, fine-tuning glucose homeostasis. The interplay between these hormones ensures that blood glucose levels remain within a narrow range, providing a steady body energy supply^[11,12]^. The adrenal glands atop the kidneys produce several hormones critical for endocrine regulation, including glucocorticoids, mineralocorticoids, and catecholamines. Glucocorticoids like cortisol are involved in the stress response, metabolism, and immune regulation. Mineralocorticoids like aldosterone regulate sodium and potassium balance and blood pressure by acting on the kidneys to promote sodium reabsorption and potassium excretion. Catecholamines, including epinephrine and norepinephrine, are released in response to stress. They exert their effects by binding to adrenergic receptors in various tissues, leading to increased heart rate, blood pressure, and energy mobilization^[13,21]^. The interaction between the endocrine and other organ systems also regulates cardiovascular function. Hormones like adrenaline, noradrenaline, and angiotensin II are critical in regulating heart rate, vascular tone, and blood pressure. Additionally, atrial natriuretic peptide (ANP), produced by the heart in response to increased blood volume, acts on the kidneys to promote sodium excretion, thereby reducing blood pressure and volume. The endocrine system also influences vascular health through insulin, which promotes vasodilation and anti-inflammatory effects, and thyroid hormones, which regulate cardiovascular function by influencing heart rate and contractility^[14,15]^. The gastrointestinal (GI) tract also produces several hormones that regulate digestive processes and energy balance. Gastrin, secreted by the stomach, stimulates the release of gastric acid, which is essential for digestion. Cholecystokinin (CCK), produced by the small intestine, promotes the release of digestive enzymes from the pancreas and bile from the gallbladder, facilitating the digestion of fats and proteins. Ghrelin, produced by the stomach, not only stimulates appetite but also promotes gastric motility and growth hormone release. Conversely, peptide YY (PYY) and glucagon-like peptide-1 (GLP-1), released by the intestines in response to food intake, inhibit appetite and slow gastric emptying, contributing to satiety and the regulation of energy balance^[16,17]^. In addition to its traditional roles, recent research has highlighted the endocrine system’s involvement in regulating bone metabolism and maintaining skeletal health. Parathyroid hormone (PTH) and vitamin D are key calcium and phosphate homeostasis regulators, essential for bone mineralization. PTH increases blood calcium levels by promoting calcium release from bones, reabsorption in the kidneys, and activation of vitamin D. Vitamin D, in turn, enhances calcium absorption from the intestines. Calcitonin, produced by the thyroid gland, opposes PTH by inhibiting bone resorption and lowering blood calcium levels. The balance between these hormones is crucial for maintaining bone density and preventing disorders like osteoporosis^[18,19]^. The endocrine system also plays a significant role in regulating reproductive health and fertility. In females, the menstrual cycle is controlled by a complex interplay of hormones, including estrogen, progesterone, LH, and FSH, which regulate the growth and release of the ovum, the preparation of the endometrium for implantation, and the maintenance of pregnancy. In males, testosterone, produced by the testes, is essential for spermatogenesis, the development of male secondary sexual characteristics, and libido. The regulation of reproductive hormones is tightly controlled by feedback mechanisms involving the hypothalamus, pituitary gland, and gonads, ensuring that reproductive processes occur in a coordinated and timely manner^[20,22]^. Lastly, the endocrine system’s role in regulating metabolism extends to controlling lipid and protein metabolism. Insulin, growth hormone, and thyroid hormones play central roles in these processes. Insulin promotes the storage of lipids in adipose tissue and the synthesis of proteins in muscle, while growth hormone stimulates lipolysis and protein synthesis, promoting growth and development. Thyroid hormones regulate basal metabolic rate, influencing the breakdown of lipids and the synthesis of proteins. Dysregulation of these hormones can lead to metabolic disorders, such as obesity, hyperlipidemia, and muscle wasting^[24,25]^.

## Current perspectives

**Figure 11.**
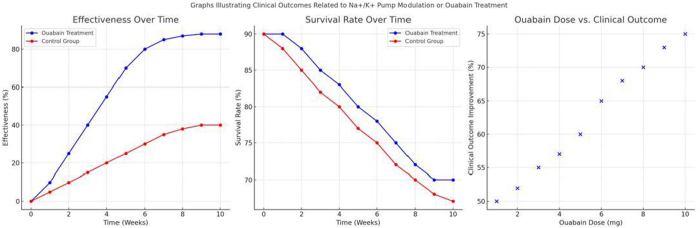
Clinical outcomes of Na^+^/K^+^ pump modulation and Ouabain treatment: effectiveness, survival rates, and dose-response relationships. A. Effectiveness over time: a line graph comparing the effectiveness of Ouabain treatment vs. a control group. B. Survival rate over time: a line graph showing survival rates in patients treated with Ouabain compared to a control group. C. Ouabain dose vs. clinical outcome: a scatter plot showing the relationship between Ouabain dose and clinical outcome improvement. Source: Authors’ creations.

**Figure 12.**
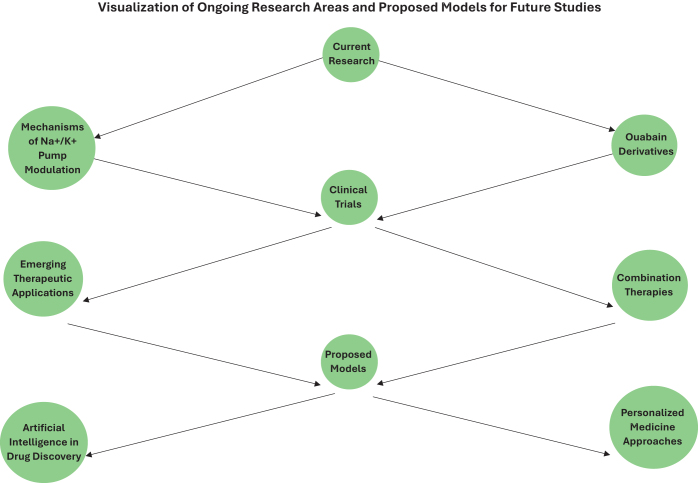
Visualization of ongoing research areas and proposed models for future studies.

Recent studies have underscored the Na^+^/K^+^ pump’s role in metabolic regulation and immune system modulation, revealing new dimensions of its physiological importance. Ouabain, once thought to be an exogenous compound derived from plants, is now known to be an endogenous hormone produced by the adrenal glands. However, this assertion remains a subject of scientific debate^[38]^. While early studies supported the adrenal origin of endogenous Ouabain, some subsequent investigations failed to detect significant levels in human plasma, raising questions about its biosynthesis and physiological role. Recent advances in high-resolution mass spectrometry and immunoassay techniques have provided stronger evidence supporting the presence of endogenous Ouabain in adrenal cortical cells. However, alternative hypotheses suggest the existence of structurally similar cardiotonic steroids with overlapping functions. This discovery has led to a paradigm shift, positioning the Na^+^/K^+^ pump at the center of a complex signaling network that influences numerous physiological processes, including cardiovascular regulation, cell proliferation, apoptosis, and immune responses^[39]^. The binding of Ouabain to the Na^+^/K^+^ pump initiates a cascade of intracellular signaling events that involve the activation of mitogen-activated protein kinases (MAPKs), phosphoinositide 3-kinase (PI3K), and the generation of reactive oxygen species (ROS). Recent investigations have elucidated the intricate relationship between the Na^+^/K^+^ pump and inflammation, showing that Ouabain can modulate pro-inflammatory and anti-inflammatory pathways through nuclear factor-kappa B (NF-κB) and Janus kinase/signal transducer and activator of transcription (JAK/STAT) signaling. These pathways are implicated in various physiological and pathophysiological processes, such as hypertension, heart failure, cancer, and autoimmune diseases^[1-3]^. In the context of immune system modulation, contemporary research highlights the Na^+^/K^+^ pump’s role in regulating immune cell activation and cytokine production. Recent evidence suggests that Ouabain can inhibit the secretion of pro-inflammatory cytokines, such as tumor necrosis factor-alpha (TNF-α) and interleukin-6 (IL-6), in macrophages, thereby mitigating inflammatory responses in conditions like rheumatoid arthritis and multiple sclerosis. Conversely, in specific pathological conditions, Ouabain-induced signaling enhances the production of ROS, thereby exacerbating inflammation. Understanding these dual effects is critical for leveraging the Na^+^/K^+^ pump as a therapeutic target for inflammatory and autoimmune diseases^[4-6]^. The involvement of the Na^+^/K^+^ pump in cancer biology represents another growing interest. Alterations in Na^+^/K^+^ pump activity have been linked to various types of cancer, with evidence suggesting that the pump’s signaling functions play a critical role in tumorigenesis. Ouabain and other cardiac glycosides have been shown to influence cell proliferation, apoptosis, and migration in cancer cells, often dose-dependent. A recent meta-analysis of oncological studies identified a correlation between Na^+^/K^+^ pump dysregulation and increased metastatic potential in lung and breast cancers, further emphasizing its significance as a biomarker and therapeutic target. For example, low concentrations of Ouabain have been found to promote cell proliferation through the activation of MAPKs and PI3K. In contrast, higher concentrations induce apoptosis by increasing intracellular sodium and calcium levels^[7-9]^. Recent analyses have further clarified the therapeutic potential of Ouabain, particularly in terms of its dose-dependent efficacy, survival benefits, and sustained clinical outcomes when compared to conventional treatment strategies. These findings are illustrated in Fig. 11, which shows comparative graphs of treatment effectiveness, survival rates, and dose-response correlations based on recent clinical data. Emerging research on the Na^+^/K^+^ pump’s impact on metabolic regulation has also gained momentum. A novel study using transcriptomic profiling revealed that Ouabain-sensitive Na^+^/K^+^ pump signaling influences glucose metabolism by modulating insulin receptor pathways. These findings suggest potential therapeutic applications in managing type 2 diabetes and metabolic syndrome. The pump’s activity is a major consumer of cellular ATP, linking it to the cell’s energy metabolism. Furthermore, Na^+^/K^+^ pump-mediated metabolic control may extend to lipid homeostasis, with recent studies demonstrating that Ouabain treatment alters lipid oxidation and mitochondrial function in adipocytes and hepatocytes^[10-12]^. Despite significant advances in understanding the Na^+^/K^+^ pump as an endocrine receptor, the debate surrounding endogenous Ouabain remains unresolved. Recent proteomic analyses utilizing high-resolution mass spectrometry have provided novel evidence supporting the presence of endogenously synthesized Ouabain in adrenal cortical cells, reigniting this debate. Some researchers argue that what has been identified as endogenous Ouabain may be structurally related but distinct cardiotonic steroids, such as marinobufagenin or telocinobufagin. These compounds share functional similarities with Ouabain and may contribute to Na^+^/K^+^ pump modulation in physiological and pathological contexts^[40,41]^. The controversy persists largely due to technical challenges distinguishing these molecules from existing detection methods, leading to conflicting findings. An active research area is developing new drugs that selectively modulate the Na^+^/K^+^ pump’s signaling functions without disrupting its essential ion transport activity. Additionally, identifying biomarkers that can predict patient responses to therapies targeting the Na^+^/K^+^ pump could enhance the precision and efficacy of these treatments. As understanding the Na⁺/K⁺ pump’s role in signaling and systemic regulation continues to evolve, new research and therapeutic application avenues are being recognized. Fig. 12 summarizes emerging concepts and outlines priority areas for future investigation in this expanding field. A 2023 clinical trial investigating Na^+^/K^+^ pump-targeted small molecules demonstrated promising results in attenuating inflammatory markers in rheumatoid arthritis patients, opening new avenues for therapeutic interventions. Advances in molecular, structural, and systems biology have provided new tools for studying the Na^+^/K^+^ pump at a molecular level, revealing details about its structure, function, and regulation^[41-43]^.

## Concluding remarks

The evolution of the Ouabain-Na^+^ pump endocrine system concept over the past 40 years has redefined its role from a simple ion transporter to a crucial regulator of cellular physiology and homeostasis. This transformation has illuminated its significance in blood pressure regulation, cellular signaling, and ion homeostasis, establishing it as a key integrator of physiological processes. The integration of Ouabain and the Na^+^/K^+^ pump within an endocrine framework underscores their broader influence on systemic health, highlighting potential implications for disease pathophysiology and therapeutic interventions. Beyond its fundamental role in maintaining ion gradients, emerging evidence suggests that dysregulation of the Na^+^/K^+^ pump contributes to conditions such as hypertension, heart failure, neuropsychiatric disorders, and even cancer. This expanding understanding paves the way for novel therapeutic strategies that target the pump’s activity through pharmacological modulation or personalized medicine approaches. By bridging basic cellular mechanisms with complex disease states, the Na^+^/K^+^ pump-endocrine system concept provides a powerful framework for future research and clinical applications. Ultimately, this concept highlights the intricate interplay between cellular and systemic physiology, reinforcing the importance of cross-disciplinary exploration in uncovering new therapeutic pathways. Just as the “Pump” sustains cellular function and the “Exchanger” facilitates dynamic balance, the metaphorical “Holy Spirit” represents the unseen but profound regulatory forces that shape health and disease – recognizing the Na^+^/K^+^ pump as more than a passive ion transporter but a central player in integrated physiological networks opens new frontiers for scientific inquiry and medical innovation.

## Call to action

The evolving concept of the Ouabain-Na^+^/K^+^ pump as an endocrine regulator presents a compelling opportunity for further research and clinical innovation. To fully harness its therapeutic potential, interdisciplinary collaboration is essential, bringing together researchers in physiology, pharmacology, genetics, and clinical medicine. Future studies should prioritize:

- Deciphering the genetic and epigenetic factors influencing the Na^+^/K^+^ pump and endogenous Ouabain to enable personalized therapeutic strategies.
- Expanding translational research to explore targeted pharmacological modulators for conditions such as hypertension, neuropsychiatric disorders, and cancer.
- Investigating the Na^+^/K^+^ pump’s role in aging and its implications for health span and longevity.
- Enhancing clinical trials to validate Na^+^/K^+^ pump-targeted therapies and integrate them into evidence-based medical practice.

By advancing research in these areas, scientists and clinicians can unlock new frontiers in understanding cellular physiology and disease treatment. The Na^+^/K^+^ pump-endocrine system concept is more than a theoretical model – it is a gateway to novel therapeutic pathways that could revolutionize modern medicine. Now is the time to expand its exploration, refine its applications, and translate these insights into tangible health benefits for patients worldwide.

## Data Availability

This published article and its supplementary information files include all data generated or analyzed during this study.
